# Multispecies biofilm behavior and host interaction support the association of *Tannerella serpentiformis* with periodontal health

**DOI:** 10.1111/omi.12385

**Published:** 2022-08-29

**Authors:** Fabian L. Kendlbacher, Susanne Bloch, Fiona F. Hager‐Mair, Johanna Bacher, Bettina Janesch, Thomas Thurnheer, Oleh Andrukhov, Christina Schäffer

**Affiliations:** ^1^ NanoGlycobiology Unit, Department of NanoBiotechnology Universität für Bodenkultur Wien Vienna Austria; ^2^ Clinic of Conservative and Preventive Dentistry Division of Clinical Oral Microbiology and Immunology Center of Dental Medicine University of Zürich Zürich Switzerland; ^3^ Competence Center for Periodontal Research University Clinic of Dentistry, Medical University of Vienna Vienna Austria

**Keywords:** biofilm composition and architecture, cell adhesion and invasion, immunostimulatory potential, multispecies model biofilm, periodontitis, *Tannerella* species

## Abstract

The recently identified bacterium *Tannerella serpentiformis* is the closest phylogenetic relative of *Tannerella forsythia*, whose presence in oral biofilms is associated with periodontitis. Conversely, *T. serpentiformis* is considered health‐associated. This discrepancy was investigated in a comparative study of the two *Tannerella* species. The biofilm behavior was analyzed upon their addition and of *Porphyromonas gingivalis—*each bacterium separately or in combinations—to an in vitro five‐species oral model biofilm. Biofilm composition and architecture was analyzed quantitatively using real‐time PCR and qualitatively by fluorescence in situ hybridization/confocal laser scanning microscopy, and by scanning electron microscopy. The presence of *T. serpentiformis* led to a decrease of the total cell number of biofilm bacteria, while *P. gingivalis* was growth‐promoting. This effect was mitigated by *T. serpentiformis* when added to the biofilm together with *P. gingivalis*. Notably, *T. serpentiformis* outcompeted *T. forsythia* numbers when the two species were simultaneously added to the biofilm compared to biofilms containing *T. forsythia* alone. *Tannerella serpentiformis* appeared evenly distributed throughout the multispecies biofilm, while *T. forsythia* was surface‐located. Adhesion and invasion assays revealed that *T. serpentiformis* was significantly less effective in invading human gingival epithelial cells than *T. forsythia*. Furthermore, compared to *T. forsythia*, a higher immunostimulatory potential of human gingival fibroblasts and macrophages was revealed for *T. serpentiformis*, based on mRNA expression levels of the inflammatory mediators interleukin 6 (IL‐6), IL‐8, monocyte chemoattractant protein‐1 and tumor necrosis factor α, and production of the corresponding proteins. Collectively, these data support the potential of *T. serpentiformis* to interfere with biological processes relevant to the establishment of periodontitis.

## Graphical abstract

Recently identified *Tannerella serpentiformis* is the closest phylogenetic relative of the periodontal pathogen *Tannerella forsythia*. First comparative biological studies support the health‐association of *T. serpentiformis* and its potential to outcompete *T. forsythia* and *Porphyromonas gingivalis* in oral biofilms.

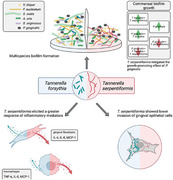

**Abbreviations**
A. *oris*
: *Actinomyces* oris
BHI: brain heart infusion
CBA: Columbia Blood Agar
CBB: colloidal Coomassie Brilliant Blue R‐250
CFU: colony forming unit
CLSM: confocal laser scanning microscopy
Cq: quantification cycle
DMEM: Dulbecco’s Modified Eagle’s Medium
F. *nucleatum*
: *Fn, Fusobacterium nucleatum*
FA: Fastidious Anaerobe agar
FAB: Fastidious Anaerobe Broth
FBS: fetal bovine serum
FISH: fluorescence in situ hybridization
HA: hydroxyapatite
hGFBs: human gingival fibroblasts
IL: interleukin
MCP‐1: monocyte chemoattractant protein‐1
MEM: minimal essential medium
mFUM: modified fluid universal medium
MOI: multiplicity of infection
MTT: 3‐(4,5‐dimethylthiazol‐2‐yl)‐2,‐diphenyltetrazolium bromide
NAMA: *N*‐acetylmuramic acid
OD: optical density
*P. gingivalis*
: *Pg, Porphyromonas gingivalis*
PAS: periodic acid Schiff reagent
PBS: phosphate‐buffered saline
Pen‐Strep: penicillin‐streptomycin
PFA: paraformaldehyde
qRT‐PCR: quantitative real‐time polymerase chain reaction
*S. anginosus*
: *Streptococcus anginosus*
SD: standard deviation
SDS‐PAGE: sodium dodecylsulfate polyacrylamide gel electrophoresis
s.e.m.: standard error of the mean
SEM: scanning electron microscopy
*S. oralis*
: *Streptococcus oralis*
*T. forsythia*
: *Tf, Tannerella forsythia*
*T. serpentiformis*
: *Ts, Tannerella serpentiformis*
TNF α: tumor necrosis factor (TNF) α
*V. dispar*
: *Veillonella dispar*

## 1 INTRODUCTION

The coexistence of the oral microbiota and the human host is characterized by networks of synergistic and antagonistic interactions that generate microbial interdependencies and provide microbial biofilms and the host immune system with a resilience to minor environmental perturbations, thereby contributing to oral health. If key environmental pressures exceed individual thresholds associated with health, the competitiveness among oral microbes is altered and dysbiosis can occur which increases the risk of periodontal diseases (Ebersole et al., 2016; Marsh & Zaura, 2017; Naginyte et al., 2019). Periodontitis is an inflammatory, polymicrobial biofilm disease of the periodontium that is clinically characterized by gingival bleeding, alveolar bone resorption, and might result in tooth loss if untreated (Kinane et al., 2017). There is evidence for a relationship between periodontitis and numerous systemic conditions, including cardiovascular disease, diabetes, cancer, rheumatoid arthritis, and Alzheimer's disease, among others (Dominy et al., 2019; Hajishengallis, 2015; Park et al., 2019). Although the role of specific bacteria in the initiation and progression of periodontitis remains debatable (Bartold & Van Dyke, 2019), the disease is associated with an increased prevalence of the “red‐complex” of Gram‐negative anaerobes—*Porphyromonas gingivalis*, *Treponema denticola*, and *Tannerella forsythia*—acting as late colonizers of the polymicrobial biofilm (dental plaque) that adheres to the surface of the teeth (Darveau, 2010; Hajishengallis, 2014; Holt & Ebersole, 2005; Socransky et al., 1998; Trindade et al., 2014). The “red complex” bacteria have synergistic relationships (Bloch et al., 2017a; Metzger et al., 2009; Ng et al., 2019; Orth et al., 2011; Y. Zhu et al., 2013), interact with preceding biofilm colonizers (Polak et al., 2012; Socransky et al., 1998) and host tissues and immune cells (Abdi et al., 2017; de Andrade et al., 2019), and orchestrate the virulence of the microbial consortium from within the biofilm. While most previous studies were focused on *P. gingivalis* (Fiorillo et al., 2019; Hajishengallis & Diaz, 2020) showing that this bacterium can subvert the host immune system and acts as a keystone pathogen, our understanding of *T. forsythia* is currently unravelling (Bloch et al., 2019).

*Tannerella forsythia* is covered with a unique, 22‐nm thick cell surface (S‐) layer in which the glycosylated S‐layer proteins TfsA and TfsB align into a 2D‐paracrystalline lattice (Oh et al., 2013; Sekot et al., 2012). The bacterium's cell surface composition and multiple copies of an *O*‐linked S‐layer decasaccharide (Posch et al., 2011; Tomek et al., 2018) as the immediate intraspecies and bacterium–host interface have been shown to be pivotal to the establishment of *T. forsythia* in the oral biofilm community, especially to its co‐aggregation with *P. gingivalis* (Bloch et al., 2017a) as well as its recognition by the immune system in a macrophage cell culture model (Sekot et al., 2011). The S‐layer delays the recognition of the bacterium at the early phase of infection (Sekot et al., 2011) and modulates dendritic cell effector functions in a way that glycosylation ensures the persistence of the pathogen in the host (Settem et al., 2013; Tomek et al., 2018). Furthermore, S‐layer deficient *T. forsythia* lost its adherence ability to human gingival epithelial cells (Ca9‐22) translating into decreased infectivity (Sakakibara et al., 2007). All together, these data support the role of the glycosylated S‐layer of *T. forsythia* as a virulence factor. Importantly, due to its auxotrophy for the essential peptidoglycan cell wall sugar *N*‐acetylmuramic acid, *T. forsythia* is reliant on a multispecies biofilm lifestyle in the oral cavity for scavenging of peptidoglycan turn‐over products from cohabiting bacteria (Hottmann et al., 2021, 2018).

Recently, the Gram‐negative oral anaerobe *Tannerella serpentiformis* (previously, *Tannerella* HOT‐286; phylotype BU036) was affiliated as a second species to the genus *Tannerella* (Ansbro et al., 2020; Frey et al., 2018) and thus is the closest phylogenetic relative of the pathogen *T. forsythia*. *Tannerella serpentiformis* was originally isolated form subgingival plaque of a female subject with chronic periodontitis but is considered a periodontal health‐associated bacterium (Vartoukian, Moazzez, et al., 2016). This might be supported by the absence of several virulence‐associated genes in the *T. serpentiformis* genome in comparison to the *T. forsythia* (Beall et al., 2018). These include, among others, those coding for the BspA surface antigen (Onishi et al., 2008; Sharma et al., 1998), KLIKK proteases, and sialidase NanH (Beall et al., 2018; Stafford et al., 2012). Comparable to *T. forsythia*, this novel *Tannerella* species possesses a glycosylated S‐layer of ∼24 nm thickness that is constituted by the two high‐molecular mass proteins TssA and TssB, which, share 52% and 59% amino acid identity with the S‐layer proteins of *T. forsythia* (TfsA, TfsB). Based on the gene content of a glycosylation gene cluster on the *T. serpentiformis* genome, the glycosylation of TssA and TssB is different from that of *T. forsythia* (Tomek et al., 2018; Zwickl et al., 2020). *Tannerella serpentiformis* was initially cultured together with the helper strains *Propionibacterium acnes* or *Prevotella intermedia* (Vartoukian, Moazzez, et al., 2016) until it was shown that *T. serpentiformis* monospecies growth can be stimulated by supplementation of the cultivation medium with *N*‐acetylmuramic acid, as seen with *T. forsythia*. Contrary to the rod‐shaped morphology of *T. forsythia* (Hottmann et al., 2018; Mayer et al., 2020), the bacterium displays a distinctive snake‐like, segmented morphology of about 20–40 μm length and 1 μm across (Frey et al., 2018; Züger et al., 2007).

Currently, nothing is known about a possible influence of *T. serpentiformis* on the polymicrobial oral biofilm associated with periodontitis. The present study aimed at investigating the biofilm “lifestyle” of *T. serpentiformis* and comparing the behavior of the two *Tannerella* species in the microbial biofilm consortium as well as their interaction with different types of host cells that are important to the establishment and progression of periodontitis. Specifically, a five‐species oral biofilm based on the “Zurich biofilm model” (Ammann et al., 2012) including four early colonizers and the bridging bacterium *Fusobacterium nucleatum* was used, into which *T. serpentiformis*, *T. forsythia*, and *P. gingivalis—*which is known for its impact on *T. forsythia*—was incorporated, either separately, each, or in combinations to unravel putative synergistic or antagonistic relationships. The biofilms were evaluated for their composition based on cell numbers and the localization of the individual species using quantitative real‐time PCR and fluorescence in situ hybridization/confocal laser scanning microscopy, respectively, as well as imaged by scanning electron microscopy (SEM). Furthermore, the cross‐reactivity of anti‐*T. forsythia* S‐layer antibodies with the *T. serpentiformis* S‐layer was investigated. To obtain a first insight into potential differences in the interaction of *T. serpentiformis* and *T. forsythia* with host cells, their adhesion to and invasion of gingival epithelial cells was investigated and their potential to induce the production of the pro‐inflammatory mediators interleukin (IL)‐1β, IL‐6, IL‐8, monocyte chemoattractant protein (MCP)‐1, and tumor necrosis factor (TNF)‐α by macrophages and primary human gingival fibroblasts was assessed.

## 2 METHODS

### 2.1 General methods

SDS‐PAGE of *T. serpentiformis* and *T. forsythia* cells was performed according to Laemmli using a Mini‐Protean II electrophoresis apparatus (Bio‐Rad, Hercules, CA, USA) (Laemmli, 1970). Carbohydrates on separated protein bands were stained with the periodic acid Schiff reagent (PAS) (Doerner & White, 1990), and protein bands were visualized with colloidal Coomassie Brilliant Blue R‐250 (CBB; Serva, Heidelberg, Germany) or transferred onto a polyvinylidene difluoride membrane (Bio‐Rad) for Western‐blot analysis. Polyclonal rabbit antisera raised against the recombinant *T. forsythia* S‐layer proteins TfsA (α‐TfsA) and TfsB (α‐TfsB) (Sekot et al., 2012) were used as primary antibodies in combination with a monoclonal goat α‐rabbit IgG secondary antibody labeled with IRDye 800CW (LI‐COR Biosciences, Lincoln, NE, USA). Bands were visualized at 800 nm using an Odyssey Infrared Imaging System (LI‐COR Biosciences). Protein concentrations were determined using the Bradford Assay Kit (Bio‐Rad) (Bradford, 1976).

### 2.2 *Tannerella* species and cultivation conditions

*Tannerella serpentiformis* W11667 (kindly provided by Dr. Graham Stafford, Integrated BioSciences, School of Clinical Dentistry, University of Sheffield, UK) was grown anaerobically on Fastidious Anaerobe agar (FA; Lab M), supplemented with 5% horse blood (Sigma–Aldrich, Darmstadt, Germany) and 20 μg/ml *N*‐acetylmuramic acid (NAMA; Sigma–Aldrich) (Vartoukian, Adamowska, et al., 2016) for 7–10 days at 37°C. Subsequently, the biomass was scraped from the plate and transferred into 10 ml of Fastidious Anaerobe Broth (FAB, E&O Laboratories Ltd., Bonnybridge, UK) or brain heart infusion (BHI) broth (Oxoid, Basingstoke, UK) at 37°C prior to further use. *Tannerella forsythia* ATCC 43037 (American Type Culture Collection, Manassas, VA, USA) was grown anaerobically on FA‐NAMA‐blood agar as above prior to liquid cultivation for 24 h at 37°C in BHI broth. All liquid media had the same supplements as the agar media (*i.e*., 20 μg/ml NAMA and 5% horse serum).

Growth curves of *T. serpentiformis* and *T. forsythia* in 10 ml of FAB and BHI broth, respectively, were recorded over a time of 160 h. For this purpose, 1 ml of an over‐night liquid culture was inoculated, the OD_600_ of the inoculum was set to 0.1 with medium, and bacterial growth was monitored by OD_600_ measurement in three independent experiments with three technical replicates, each. The growth rate *μ* [h^–1^] and the doubling time *t_d_* [h] of the bacteria in the exponential phase were calculated according to *μ* = (lnOD_1_‐lnOD_2_)/(*t*2‐*t*1) and *t_d_ *= ln2/*μ*.

### 2.3 Growth of *Tannerella* species biofilms in polystyrene plates

Biofilm growth of *T. serpentiformis* and *T. forsythia* in polystyrene plates was determined by differential OD_600_ measurement of resuspended biofilm versus total bacterial culture. For this purpose, half‐concentrated BHI medium (diluted with 1× PBS) (Friedrich et al., 2017) with supplements as described above was inoculated with either *T. forsythia* or *T. serpentiformis*, each grown on FA blood agar plates supplemented with NAMA (10 μg/ml), and set to an OD_600_ of 0.05 with growth medium. The bacteria were grown anaerobically at 37°C for 6 days in 24‐well polystyrene plates coated with mucin (from bovine submaxillary gland, Sigma–Aldrich, St. Louis, USA) solution (0.5 mg/ml in 0.1 M sodium acetate buffer, pH 4.5). One ml of cell suspension was added to five wells, each, per strain, sterile half‐concentrated BHI medium served as a negative control. Two wells per strain were used to determine the total OD_600_ for normalization. From the other wells, medium and planktonic cells were removed and the remaining cells forming a biofilm washed gently with 500 μl distilled H_2_O and finally resuspended in 1 ml distilled H_2_O, and the OD_600_ of the biofilm was measured. The values were normalized to the total OD_600_ of each bacterium.

### 2.4 Growth of multispecies biofilms on hydroxyapatite discs

Five‐species biofilms based on the “Zurich subgingival biofilm model” (Ammann et al., 2012) were established. These were comprised of *Fusobacterium nucleatum* (OMZ598), *Actinomyces oris* (OMZ745), *Veillonella dispar* (OMZ493), *Streptococcus anginosus* (OMZ871), and *Streptococcus oralis* (OMZ607), to which *T. forsythia*, *T. serpentiformis* and *P. gingivalis* (OMZ925), either each bacterium separately, or different combinations of the three bacteria were added (for details of the compositions of the different biofilms see Table 1). For this purpose, precultures of *F. nucleatum*, *A. oris*, *V. dispar*, *S. anginosus*, *S. oralis*, and *P. gingivalis* were grown anaerobically at 37°C for 3 days on Columbia Blood Agar (CBA; Oxoid) supplemented with 5% horse blood. Subsequently, the bacteria were scraped off the plates and, with the exception of *P. gingivalis*, transferred into modified fluid universal medium (mFUM) (Gmür & Guggenheim, 1983); in the case of *V. dispar*, FUM was supplemented with sodium DL‐lactate (Sigma–Aldrich; 10 μg/ml), and *P. gingivalis* was transferred into BHI broth. All bacteria were grown anaerobically at 37°C for 24 h. For the two *Tannerella* species to be added to these five‐species biofilms, the overnight cultures as prepared above were used.

**TABLE 1:** Composition of the different biofilms investigated in this study

Species	Abbreviation*
“Five species”: *Fusobacterium nucleatum* OMZ598 *Actinomyces oris* OMZ745 *Veillonella dispar* OMZ493 *Streptococcus anginosus* OMZ871 *Streptococcus oralis* OMZ607	Five species
“Five species,” *Tannerella forsythia* ATCC 43037	Tf
“Five species,” *Tannerella serpentiformis*	Ts
“Five species,” *T. forsythia* ATCC 43037, *T. serpentiformis*	TF + Ts
“Five species,” *Porphyromonas gingivalis* OMZ925	Pg
“Five species,” *T. forsythia* ATCC 43037, *P. gingivalis* OMZ925	Tf + Pg
“Five species,” *T. serpentiformis*, *P. gingivalis* OMZ925	Ts + Pg
“Five species,” *T. forsythia* ATCC 43037, *T. serpentiformis*, *P. gingivalis* OMZ925	Tf + Ts + Pg

*Abbreviation as used in Figures 3 and 4.

For multispecies biofilm formation on sintered, pellicle‐coated HA discs (9 mm in diameter; Clarkson Chromatography Products, South Williamsport, USA), all bacteria from over‐night cultures were freshly inoculated into mFUM and grown for an additional 5 h. Thereafter, the OD_600_ of these cultures was adjusted to 1.0. Subsequently, the cultures were mixed at equal volumes, and 200 μl of the cell suspension was used to inoculate 1.6 ml of growth medium (960 μl pooled saliva, 160 μl horse serum [Thermo‐Fischer] and 480 μl mFUM) (Gmür & Guggenheim, 1983) in 24‐well polystyrene tissue culture plates (Greiner, Darmstadt, Germany). The medium was changed after 16 h and 40 h, and discs were dip‐washed in 0.9% NaCl twice a day. After anaerobic incubation at 37°C for 64 h, biofilms were dip‐washed again and either harvested by vigorous vortexing for 3 min in 1 ml 0.9% NaCl or fixed for 1 h at 4°C in 4% paraformaldehyde (Sigma–Aldrich) solution prior to fluorescence in situ hybridization (FISH) and SEM. To image *T. forsythia* in multispecies biofilms, 100 μl of the initial culture was added at each medium change in order to obtain enough cells for FISH‐staining.

Growth of *T. forsythia* and *T. serpentiformis* monospecies biofilms and of a dual *Tannerella* species biofilm on pellicle‐coated HA discs was carried out essentially as described above for the multispecies biofilms.

### 2.5 Quantitative analysis of biofilms

The cell number of all bacteria in the biofilms was determined by qPCR (Ammann et al., 2013). For this purpose, genomic DNA was extracted from 500 μl of harvested biofilm using the GenElute Bacterial Genomic DNA kit (Sigma–Aldrich) and analyzed on an MJ Mini Thermal Cycler and MiniOpticon Detector (Bio‐Rad) using species‐specific primers (Table 2) amplifying the 16S rRNA gene (Ammann et al., 2013).

**TABLE 2:** Primers used in this study

Organism	Sequence (5′ → 3′)	Strand	Reference
*V. dispar*	CCCGGGCCTTGTACACACCG CCCACCGGCTTTGGGCACTT	+ −	(Ammann et al., 2013)
*F. nucleatum*	CGCCCGTCACACCACGAGA ACACCCTCGGAACATCCCTCCTTAC	+ −	(Ammann et al., 2013)
*S. oralis*	ACCAGGTCTTGACATCCCTCTGACC ACCACCTGTCACCTCTGTCCCG	+ −	(Ammann et al., 2013)
*A. oris*	GCCTGTCCCTTTGTGGGTGGG GCGGCTGCTGGCACGTAGTT	+ −	(Ammann et al., 2013)
*S. anginosus*	ACCAGGTCTTGACATCCCGATGCTA CCATGCACCACCTGTCACCGA	+ −	(Ammann et al., 2013)
*P. gingivalis*	GCGAGAGCCTGAACCAGCCA ACTCGTATCGCCCGTTATTCCCGTA	+ −	(Ammann et al., 2013)
*T. forsythia* (TfsB)	ACGGAGTGAAGGACTTTGCA CAAGCCTCCACCGGTACTTT	+ −	This study
*T. forsythia*/ *T. serpentiformis* (16S rRNA)	CGAGCGATCGGATGCAAATC CAGCTTCACGGAGTCGAGTT	+ −	(Ammann et al., 2013)
*T. serpentiformis* (TssB)	TCCGTACTGATCGCTGGAGA TTAGCAGCATCGAACGTGGT	+ −	This study

To quantify the bacteria, a standard curve was generated for each bacterium using the logarithm of the quantification cycle (Cq) values of the serial dilution samples. With the concentration of the extracted genomic DNA of the biofilm samples, determined using a NanoDrop ND‐1000 spectrophotometer (Thermo Fisher Scientific, Waltham, MA, USA), and the obtained Cq values, the sample DNA concentration was calculated by interpolating from the standard curve. The cell number per biofilm was further calculated with the theoretical genome weight of the respective organism (Ammann et al., 2013). The abundance of each organism was determined in three independent biological experiments with three technical replicates per biofilm.

### 2.6 Imaging of biofilms

To assess the distribution and potential aggregation of certain bacteria within the biofilms, FISH staining was performed (Thurnheer et al., 2004) using the probe combinations listed in Table 3. The fixed biofilms on HA discs were permeabilized with lysozyme solution (7 × 10^4^ U/ml in H_2_O; Sigma–Aldrich) for 10 min at room temperature and prehybridized in hybridization buffer (0.9 M NaCl, 20 mM Tris–HCl [pH 7.5], 0.01% SDS, 30% formamide) at 46°C for 15 min, followed by 3 h of hybridization with specific oligonucleotide probes (Ammann et al., 2012). This was followed by washing in wash buffer (20 mM Tris–HCl, pH 7.5, 5 mM EDTA, 0.01% SDS, 46–70 mM NaCl) for 45 min at 48°C. To counterstain the biofilms, the discs were incubated with a solution of 10 μM Sytox Green (Invitrogen/Thermo Fisher Scientific) for 30 min at room temperature in the dark. Finally, the discs were fixed in Mowiol in confocal microscopy dishes for 24 h (Thurnheer et al., 2006).

**TABLE 3:** Probes used in this study (Ammann et al., 2012)

Probe	Organism	Sequence (5′→3′)
Tan1260b‐Cy3	*Tannerella serpentiformis*	TGCATCCGATCGCTCGGT
Pging1006‐2‐Cy3	*Porphyromonas gingivalis*	GTTTTCACCATCMGTCAT
Tfor127‐Cy5	*Tannerella forsythia*	CTCTGTTGCGGGCAGGTTAC

The architecture of the biofilms was analyzed by confocal laser scanning microscopy (CLSM) using a Leica SP‐8 microscope. Images were captured using a 100× and 40× objective and processed with Fiji software (Schindelin et al., 2012).

SEM was performed to examine the biofilm architecture and to specifically compare the monospecies biofilms of the two *Tannerella* species. The fixed biofilms were dehydrated by incubation in an ascending ethanol series of 35%, 50%, 70%, 95%, and 100% for 5 min, each. High‐vacuum secondary electron imaging was performed using an Apreo VS SEM (Thermo Fisher Scientific) at 2 kV.

Presented images are snapshots of the biofilm structures present on the HA discs, and the depicted structures represent a comprehensive collection of biofilm behavior observed during sampling.

### 2.7 Cultivation of human host cells

The immortalized human gingival cell line Ca9‐22 derived from squamous carcinoma of the gingiva was used (Japanese Collection of Research Bioresources Cell Bank, JCRB0625, Ibaraki, Japan); cells were cultured in minimal essential medium (MEM; Invitrogen, Waltham, MA, USA) supplemented with 10% fetal bovine serum (FBS), 100 U/ml penicillin, and 100 μg/ml streptomycin (Pen‐Strep; Sigma–Aldrich) at 37°C in 5% CO_2_. Cells between passages 6 and 11 were used for the experiments.

The U937 monocytic cell line was purchased from ATCC and cultured in RPMI 1640 medium (Invitrogen), supplemented with 10% FBS and Pen‐Strep at 37°C in a humidified atmosphere containing 5% CO_2_ (Friedrich et al., 2015).

Human gingival fibroblasts (hGFBs) were isolated from the gingival tissue of third molar teeth of periodontally and systemically healthy individuals extracted for orthodontic reasons (Blufstein et al., 2021; Sekot et al., 2011). The procedure was approved by the Ethics Committee of the Medical University of Vienna (EK 1079/2019, extended in 2021), and all patients gave their written consent. Gingival tissue was cut off with a scalpel, placed into Dulbecco's Modified Eagle's Medium (DMEM, Invitrogen) supplemented with 10% FBS, Pen‐Strep, shredded into small pieces, and incubated at 37°C and 5% CO_2_ for cell outgrowth. Cells between passages three and six were used for the experiments.

### 2.8 Invasion and adhesion of *T. serpentiformis* and *T. forsythia* to gingival epithelial cells

Ca9‐22 cells were seeded into 24‐well tissue culture plates at a concentration of 10^5^ cells per well. After 48 h of incubation, the cells were infected with either *T. forsythia* or *T. serpentiformis* at a multiplicity of infection (MOI) of 100 for 90 min (Megson et al., 2015).

To determine the number of intracellularly invaded bacteria, the Ca9‐22 cells were washed three times with 1× PBS and incubated for further 60 min in 500 μl MEM, supplemented with 10% FBS and Pen‐Strep (Bloch et al., 2018) to kill extracellular bacteria. The cells were washed with 1× PBS followed by addition of 200 μl of sterile, distilled H_2_O per well, and cells were scraped from the wells with a pipette tip for 1 min for physical disruption. From the cell lysate, a 10‐fold serial dilution was made, and 10 μl from each dilution step was spotted in triplicate onto a FA‐NAMA‐blood agar plate and incubated anaerobically at 37°C for 8 days in order for each invading bacterium to grow and form a colony.

This procedure was repeated without addition of Pen‐Strep to obtain numbers of total bacteria associated with Ca9‐22 cells. The difference between the total count of bacteria and the count of intracellular bacteria was considered as the numbers of extracellularly adhering bacteria. The invasion and adherence of bacteria to Ca9‐22 cells was characterized as the percentage of colony‐formung unit (CFU) counts of intracellular and extracellular bacteria in relation to CFU counts obtained for bacteria grown under the same conditions without Ca9‐22 cells.

### 2.9 Stimulation of human macrophages and human gingival fibroblasts with *Tannerella* species

Prior to stimulation with bacteria, U937 monocytes were differentiated into macrophages (Braun et al., 2022; Sekot et al., 2011). Briefly, 3 ml of cell suspension at a concentration of 10^6^ cells/ml were added to each well of a 6‐well plate and cells were stimulated with phorbol 12‐myristate 13‐acetate (Sigma–Aldrich) at a concentration of 0.2 μg/ml for 72 h.

Adherent macrophages were gently scraped, counted, and seeded in a 24‐well plate at a density of 3 × 10^5^ cells/well in 0.5 ml RPMI 1640 medium supplemented with 10% FBS and Pen‐Strep. hGFBs were seeded at a density of 5 × 10^4^ cells/well in 0.5 ml DMEM containing the same supplements. After 24 h, the medium was discarded, cells were washed once with 1× PBS, and exposed to the different bacterial stimuli at an MOI of 50. Stimulation was performed in the respective media without supplements for 4 and 24 h at 37°C and 5% CO_2_. Cells incubated in the media without bacteria and supplements were used as a negative control. After stimulation, the cell viability and inflammatory response were analyzed. At least five independent experiments with four technical replicates were performed.

### 2.10 MTT cell viability assay

After cell stimulation, 100 μl of 3‐(4,5‐dimethylthiazol‐2‐yl)‐2,5‐diphenyltetrazolium bromide (MTT) dye (5 mg/ml in PBS) was added to the cells, and the plates were incubated at 37°C for 2 h (Vistica et al., 1991). Subsequently, the medium was discarded, and 500 μl of dimethylsulfoxide was added to each well. The plates were shaken to facilitate the dissolving of formazan crystals. Controls were performed in which each bacterium was solely added. OD_570_ values were measured on a Synergy HTX multi‐mode reader (BioTek Instruments, Winooski, USA).

### 2.11 Gene expression analysis of inflammatory mediators

Cell lysis, transcription into cDNA, and qPCR were performed using the TaqMan® Gene Expression Cells‐to‐CT™ kit (Ambion/Applied Biosystems, Foster City, CA, USA) (Behm et al., 2019; Blufstein et al., 2019). The target genes were amplified using the following primers (all Applied Biosystems): TNF‐α, 99999043_m1; IL‐1β Hs01555410_m1; IL‐6, Hs00985639_m1; IL‐8, Hs00174103_m1; MCP‐1, Hs00234140_m1; GAPDH, Hs99999905_m1. qPCR was performed in paired reactions using the ABI StepOnePlus device. C_t_ values were determined for each gene, and the expression of the target gene was calculated by the 2^−ΔΔCt^ method, where ΔΔC_t_ = (C_t_^target^ – C_t_^GAPDH^) sample – (C_t_^target^ – C_t_^GAPDH^) control. Cells which were not treated with bacteria served as control. For U937 macrophages, expression of TNF‐α, IL‐1β, IL‐8, and MCP‐1 was analyzed, for hGFBs, IL‐6, IL‐8, and MCP‐1.

### 2.12 Determination of secreted cytokines and chemokines by ELISA

The concentration of the inflammatory mediators IL‐1β, IL‐6, IL‐8, MCP‐1, and TNF‐α in conditioned media was determined using uncoated ELISA kits (Invitrogen) according to the manufacturer's protocol. The sensitivity of the ELISA was 2 pg/ml for IL‐1β, IL‐6, and IL‐8, 7 pg/ml for MCP‐1, and 4 pg/ml for TNF‐α.

### 2.13 Statistical analysis

Statistical analysis of the bacterial biofilms was performed using RStudio, with results being considered statistically different at *p* < 0.05. The data were initially tested for homoscedasticity with Levene's test and for normality with the Shapiro–Wilk test. Nonhomoscedastic and normally distributed samples were compared with Welch's analysis of variance (ANOVA), followed by the Games–Howell post hoc test. Homoscedastic non‐normally distributed samples were compared using the Kruskal–Wallis test. Samples passing the initial tests for normal distribution and homogeneity of variance were tested by one‐way ANOVA, followed by Tukey's test for multiple comparisons. All data are expressed as mean ± SD. Differences in invasion and adhesion between the two *Tannerella* species were analyzed with the unpaired Student's *t*‐test using RStudio, with results being considered statistically different at *p* < 0.05. Four independent experiments with three technical replicates were performed for each assay.

The Friedman test followed by the post hoc Wilcoxon test for pairwise comparison was used to analyze statistical differences of immunological data. Statistical analysis was performed using SPSS 24.0 software (IBM, Armonk, NY, USA). All data are expressed as mean ± standard error of the mean (SEM). Significant statistical differences were considered at *p* < 0.05.

## 3 RESULTS

### 3.1 General description of *T. serpentiformis* in comparison to *T. forsythia*

**FIGURE 1:**
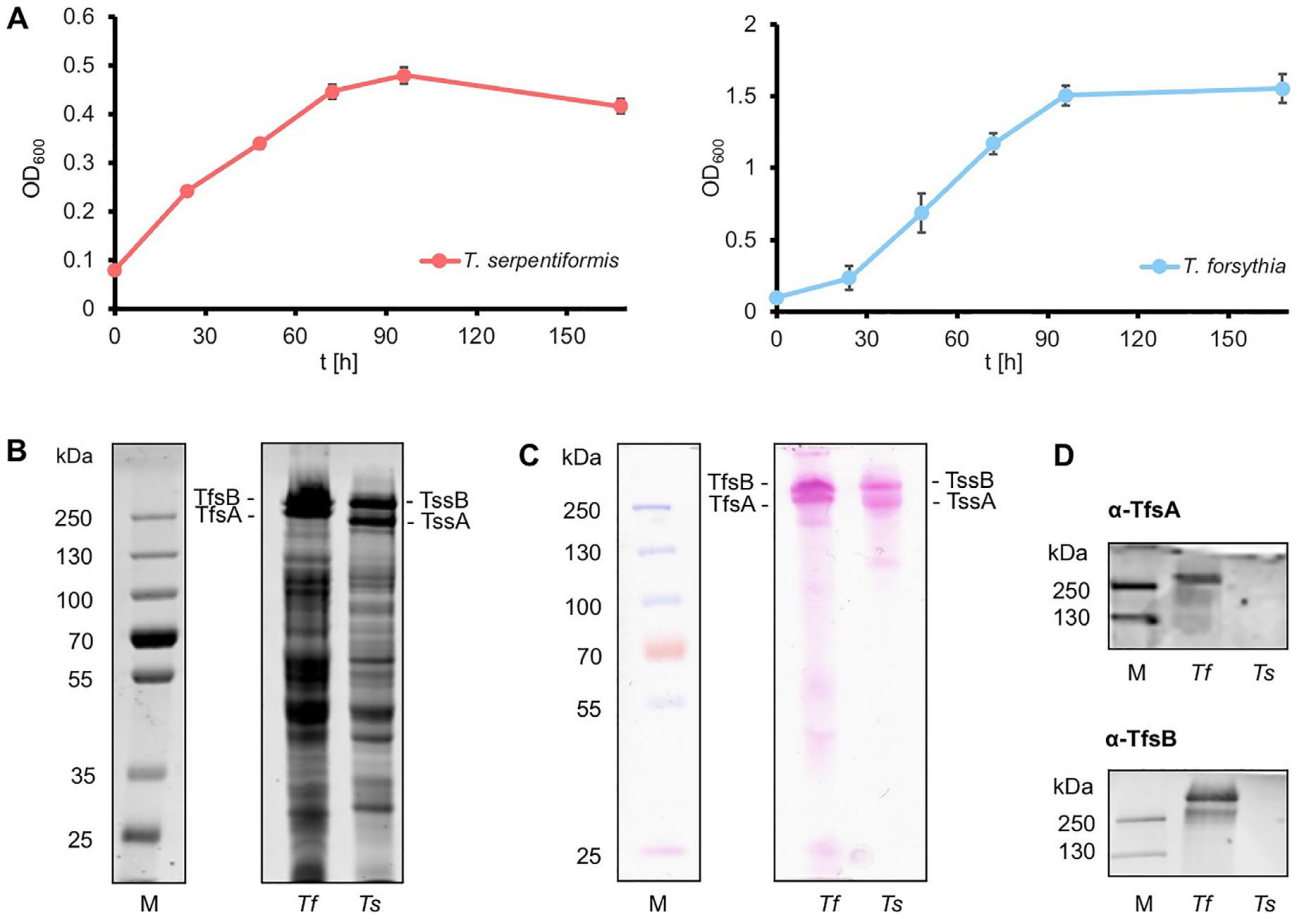
(A) Growth curve of *T. serpentiformis* (left) and *T. forsythia* (right) in liquid culture. Growth was monitored by OD_600_ measurement over 160 h.10% SDS‐PAGE of *T. forsythia* and *T. serpentiformis* cells, stained with (B) Coomassie Brilliant Blue for proteins and (C) periodic acid‐Schiff reagent for carbohydrates. (D) Western‐blot of *T. forsythia* and *T. serpentiformis* cells using the α‐TfsA and α‐TfsB antibodies raised against the *T. forsythia* S‐layer proteins. M, PageRuler Plus Prestained Protein Ladder; *Tf*, *T. forsythia*; *Ts*, *T. serpentiformis*

For *T. serpentiformis* grown in FAB, a doubling time of 37.79 ± 15.21 h (*μ* = 0.060 ± 0.022 h^‐1^) was calculated, while *T. forsythia* grew in BHI broth to higher density with a doubling time of 16.90 ± 6.52 h (μ = 0.046 ± 0.016 h^‐1^) (Figure 1a,b).

After separation of *T. serpentiformis* and *T. forsythia* biomass by SDS‐PAGE, upon staining with CBB (Figure 1b) and PAS‐reagent (Figure 1c), respectively, the high‐molecular weight S‐layer glycoproteins were clearly visible for either *Tannerella* species, with the upshift in molecular weight in comparison to the calculated molecular weight of the S‐layer proteins based on the amino acid sequence—TfsA (Tanf_03370), 133.1 kDa; TfsB (Tanf_03375), 150.4 kDa; TssA (BCB71_00675), 134.2 kDa; TssB (BCB71_00680), 156.0 kDa—in either case due to glycosylation. As previously described for *T. forsythia* (Posch et al., 2011), *T. serpentiformis* contains in addition to the prominent S‐layer glycoproteins also other glycoproteins. The *T. forsythia* S‐layer antibodies α‐TfsA and α‐TfsB showed no cross‐reactivity with the *T. serpentiformis* S‐layer proteins (Figure 1d), indicative of different cell surface‐exposed S‐layer epitopes in the two *Tannerella* species.

### 3.2 Biofilm lifestyle of *T. serpentiformis*

**FIGURE 2:**
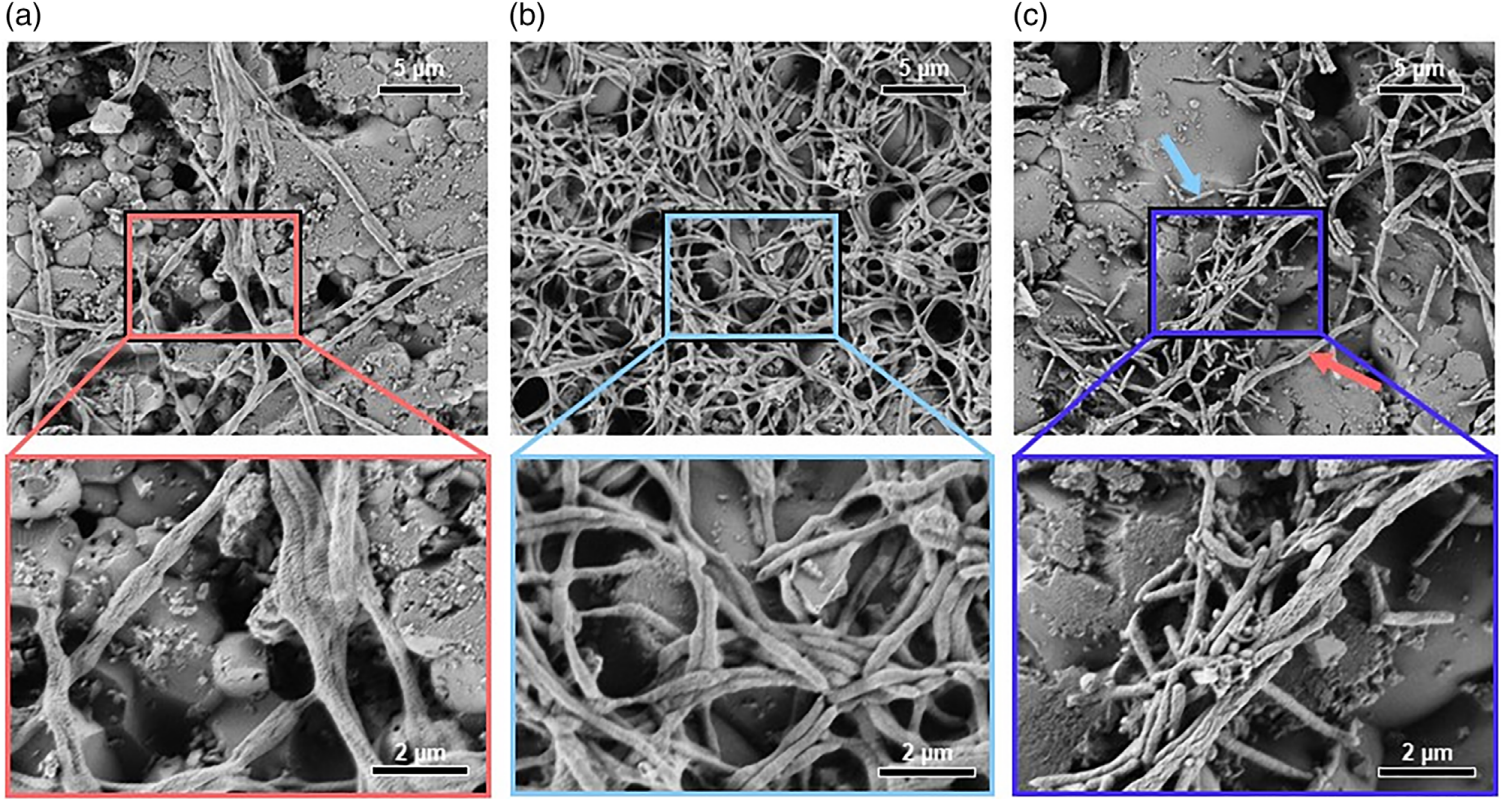
Scanning electron micrographs of *T. serpentiformis* (a) and *T. forsythia* (b) cells after biofilm growth in comparison to a mixed biofilm of the two *Tannerella* sp. (c) grown for 64 h on pellicle‐coated HA discs. *Tannerella forsythia* (blue arrow) and *T. serpentiformis* (red arrow) are discernible based on their cell morphology. The lower panel shows an enlarged view of each biofilm.

*Tannerella serpentiformis* showed a very low, if at all, capability to form a monospecies biofilm on pellicle‐coated HA discs (Figure 2a); *T. serpentiformis* cells displayed a filamentous morphology with individual rods of about 20 μm, conforming with previous data of the bacterium when grown in liquid culture (Frey et al., 2018). In contrast, *T. forsythia* formed a biofilm with a net‐like appearance and individual *T. forsythia* rods had a length of about 3 μm (Figure 2b). In the dual‐species biofilm, the two *Tannerella* species were clearly discernible based on their morphology and did not show co‐aggregation (Figure 2c). *Tannerella serpentiformis* seemed to antagonize the biofilm formation capability of *T. forsythia* in the dual‐species biofilm, as evidenced by a loose assembly of bacterial cells on the solid support. Replica of the observed biofilm structures can be found in Figure S1.

*Tannerella serpentiformis’* significantly decreased biofilm formation capability in comparison to its pathogenic counterpart was confirmed upon cultivation on mucin‐coated polystyrene plates for 6 days (Figure S2).

### 3.3 Influence of *T. serpentiformis* on a commensal oral model biofilm in comparison to *T. forsythia* and *P. gingivalis*

To analyze the effect of the two *Tannerella* species in comparison to the known keystone pathogen *P. gingivalis* on the composition of the multispecies oral biofilm, the change in the cell numbers of the commensal species (*i.e*., *A. oris*, *V. dispar*, *S. anginosus*, *S. oralis*) and of *F. nucleatum* (together termed “five species”) upon introduction of *T. forsythia*, *T. serpentiformis* and *P. gingivalis* or combinations of the three species was determined by qPCR after 64 h of incubation.

**FIGURE 3:**
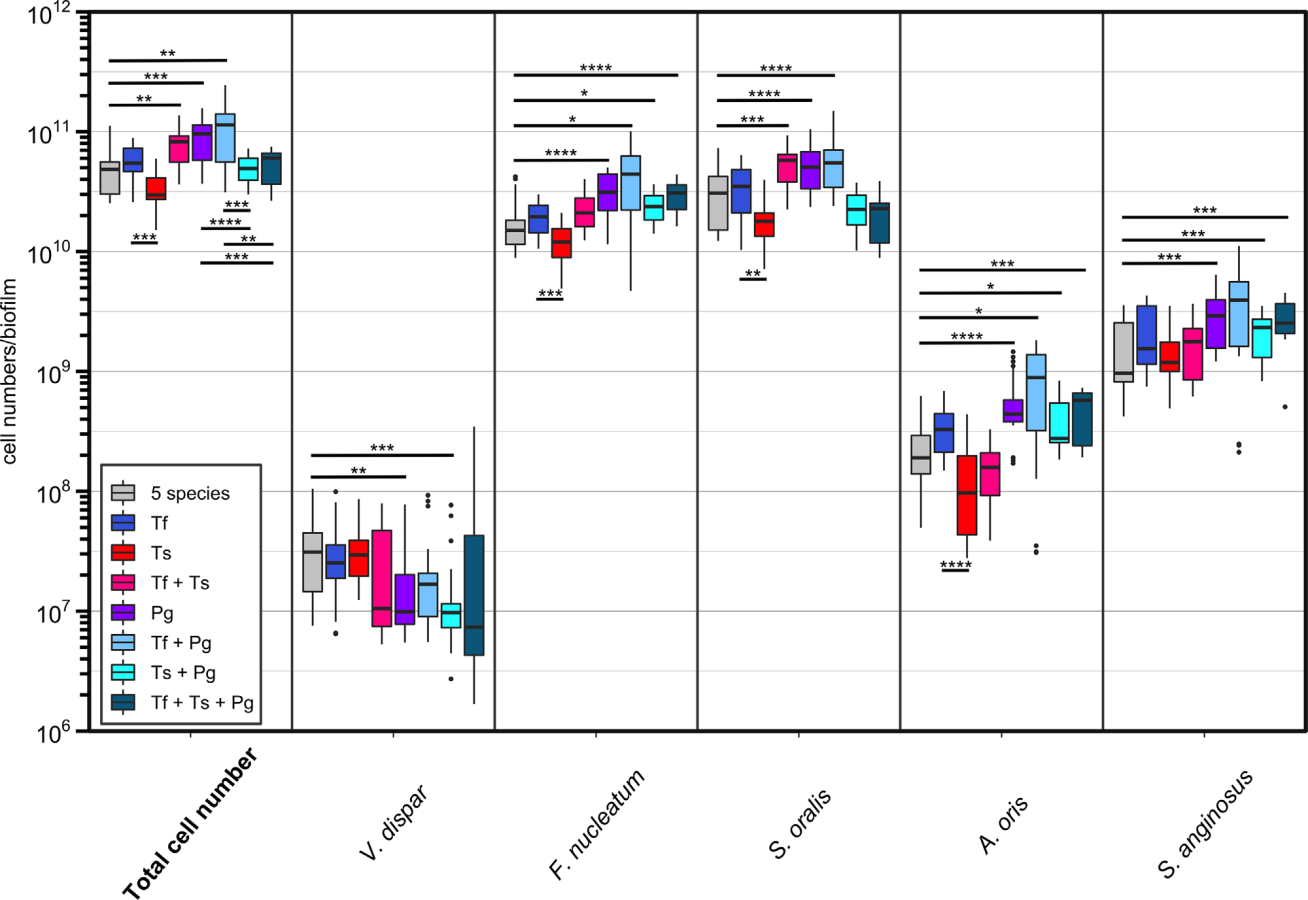
Comparison of the cell numbers of the “five species” in different biofilm set‐ups measured by qPCR. Data were obtained from three independent experiments and plotted on a logarithmic scale. Significances were determined against the commensal biofilm (five species) and are reported as *p < 0.05, **p < 0.0 1, ***p < 0.001, and ****p < 0.0001. “Five species”: *V. dispar*, *F. nucleatum*, *S. oralis*, *A. oris*, and *S. anginosus*; gray, “five species” plus *T. forsythia* (Tf) (blue), “five species” plus *T. serpentiformis* (Ts) (red), “five species” plus Tf and Ts (pink), “five species” plus *P. gingivalis* (Pg) (violet), “five species” plus Tf and Pg (light blue), five species plus Ts and Pg (turquoise), “five species” plus Tf, Ts, and Pg (petrol). For details of biofilm bacteria, see Table 1.

The total cell number of the “five species” was significantly increased upon incorporation of *P. gingivalis* (Pg), *T. forsythia* together with *P. gingivalis* (Tf + Pg), and both *Tannerella* species together (Tf + Ts) (Figure 3). In contrast, the total cell number was significantly lower in the biofilms containing *T. serpentiformis* and *P. gingivalis* (Ts + Pg or Tf + Ts + Pg) compared to the biofilms containing *T. forsythia* plus *P. gingivalis* (Tf + Pg) and *P. gingivalis* alone (Pg), supporting the growth‐mitigating effect of *T. serpentiformis*. Compared to the “five‐species” biofilm with *T. forsythia* alone, the presence of *T. serpentiformis* led to a significant decrease of the total cell number, as well as the cell numbers of *F. nucleatum*, *S. oralis*, and *A. oris*.

On the single‐species level, the number of the individual species was changed upon incorporation of *P. gingivalis* alone (Pg) or of *P. gingivalis* combined with the *Tannerella* species (Pg + Tf or Pg + Ts or Tf + Ts + Pg). *Fusobacterium nucleatum* and *A. oris* highly benefitted from the incorporation of *P. gingivalis* to the biofilm in every biofilm set‐up tested, as their cell numbers were significantly increased in all cases where *P. gingivalis* was present. This emphasizes the role of *P. gingivalis* in promoting biofilm growth. The results for *S. oralis* were similar as for the total of the “five species,” with *S. oralis* cell numbers being significantly increased in the presence of *P. gingivalis* (Pg), as well as in the presence of *T. forsythia* together with *P. gingivalis* (Tf + Pg) or *T. serpentiformis* (Tf + Ts). Interestingly, the cell number of *S. oralis*, along with the total cell number, was not significantly increased if both *T. serpentiformis* and *P. gingivalis* (Ts + Pg) were incorporated into the biofilm. Here, the presence of *T. serpentiformis* could mitigate the growth‐promoting effect of *P. gingivalis*.

Notably, *V. dispar* showed the opposite response to the addition of *P. gingivalis* (Pg) and *P. gingivalis* in combination with *T. serpentiformis* (Ts + Pg) to the biofilm with significantly reduced cell numbers, and it was detected in slightly lower cell numbers when both *Tannerella* species together with or without *P. gingivalis* where present (Tf + Ts or Tf + Ts +Pg).

### 3.4 Quantitative analysis of *T. forsythia*, *T. serpentiformis*, and *P. gingivalis* in the commensal biofilm

**FIGURE 4:**
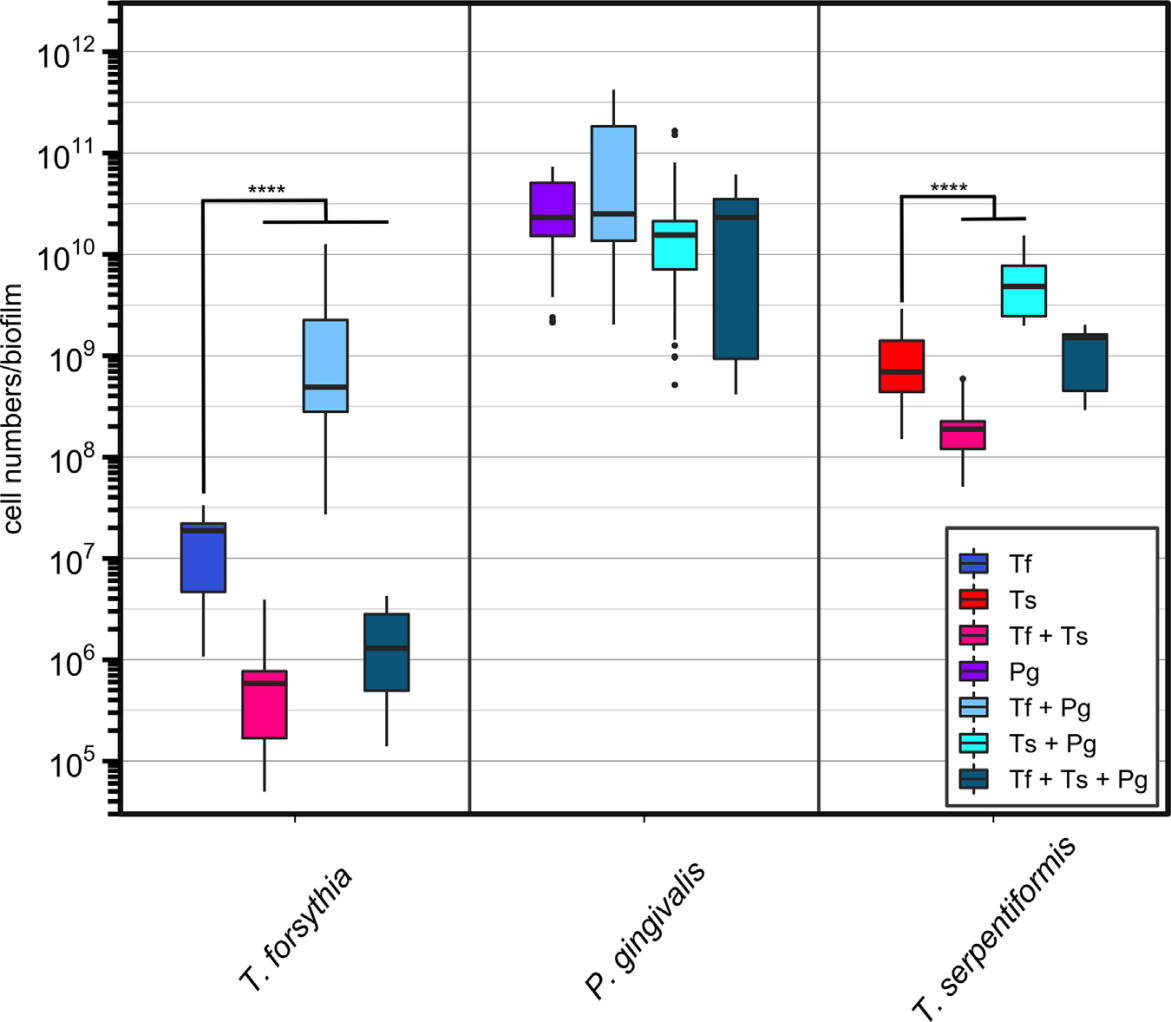
Comparison of the cell numbers of the two *Tannerella* species and *P. gingivalis* in different biofilm set‐ups according to qPCR results. Data derived from three independent experiments were plotted on a logarithmic scale. Significances were determined against the *Tannerella* biofilms (Tf and Ts) and are reported as ****p < 0.0001. “Five species”: *V. dispar*, *F. nucleatum*, *S. oralis*, *A. oris*, and *S. anginosus*. “Five species” plus *T. forsythia* (Tf) (blue), “five species” plus *T. serpentiformis* (Ts) (red), “five species” plus Tf and Ts (pink), five species plus *P. gingivalis* (Pg) (violet), “five species” plus Tf and Pg (light blue), “five species” plus Ts and Pg (turquoise), “five species” plus Tf, Ts, and Pg (petrol). For details of biofilm bacteria, see Table 1.

In addition to the “five‐species,” the cell numbers of both *Tannerella* species and of *P. gingivalis* were determined by qPCR in the same biofilms in order to assess their behavior in the commensal biofilm (Figure 4).

Similar as for the commensals, the presence of *P. gingivalis* significantly increased *T. forsythia* and *T. serpentiformis* cell numbers (Figure 4). However, this growth‐promoting effect could not be observed when both *Tannerella* species were incorporated into the biofilm in addition to *P. gingivalis*, especially for *T. forsythia*. Moreover, the two *Tannerella* species seemed to be competing, as indicated by their cell numbers being significantly decreased when both were added to the commensal biofilm. Conversely, *P. gingivalis* cell numbers were constant across the different biofilm setups, with only slight decreases in the presence of *T. serpentiformis*.

### 3.5 Imaging of the multispecies biofilm structure by CLSM and SEM

To assess potential differences in the localization of the *Tannerella* species in the multispecies biofilm, their ability to aggregate with other species, and their influence on the overall biofilm structure, FISH and subsequent CLSM analysis as well as SEM of fixed biofilms was performed for qualitative evaluation.

**FIGURE 5:**
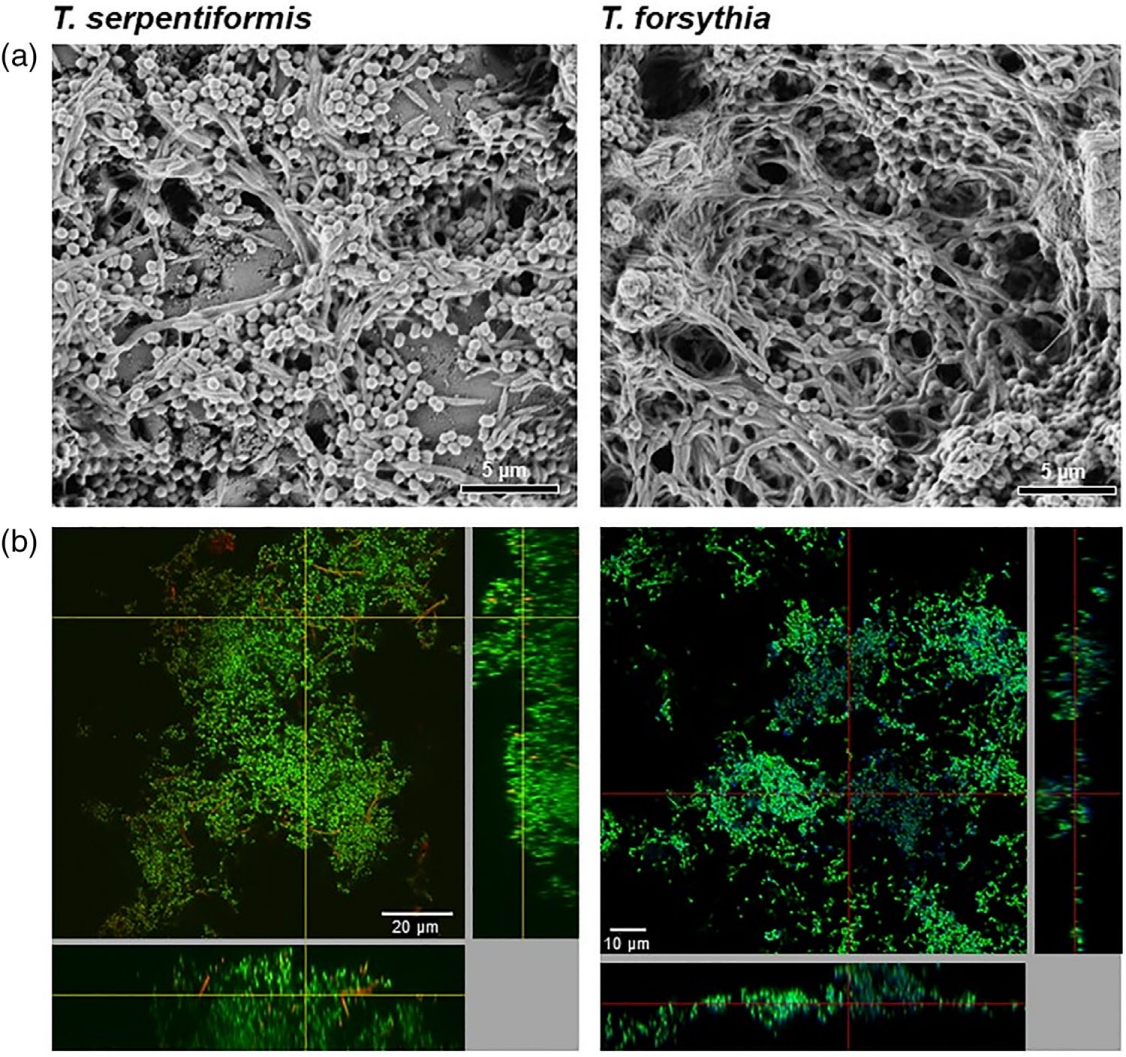
SEM micrographs (a) of a fixed, “five‐species” biofilm containing *T. serpentiformis* and *T. forsythia*, grown for 64 h on a pellicle‐coated HA disc. CLSM images (b) of the same biofilms with FISH‐probes specific for *T. serpentiformis* and *T. forsythia* for localization of the *Tannerella* species in the biofilm consortium. Red: *T. serpentiformis*; blue: *T. forsythia*; green: nonhybridized cells (DNA staining Sytox green). One area of the biofilm is presented here as viewed from the top in the big upper left panel with side views in the right and bottom panels. The biofilm surface is directed toward the top left panel. Objective 100×.

The “five‐species” biofilm containing *T. serpentiformis* (Figure 5a, left) was looser, with less coverage of the HA disc according to SEM evidence in comparison to the biofilm containing *T. forsythia* (Figure 5a, right).

In the CLSM images with FISH‐probes specific for the individual *Tannerella* species, the long, segmented *T. serpentiformis* cells could be detected as single cells throughout the whole biofilm structure (Figure 5b, left), without an indication of cluster formation. In contrast, *T. forsythia* cells were found in large groups in the upper layer of the biofilm, closely associated with itself and other bacteria (Figure 5b, right), conforming with previous data (Bloch et al., 2017b). The cells could only be detected when fresh culture of *T. forsythia* was added to the medium, thereby increasing its concentration by almost two log units to 9.83 × 10^8^ ± 2.15 × 10^8^ cells per biofilm. Replica of the biofilm structures shown in Figure 5 can be found in Figure S3.

**FIGURE 6:**
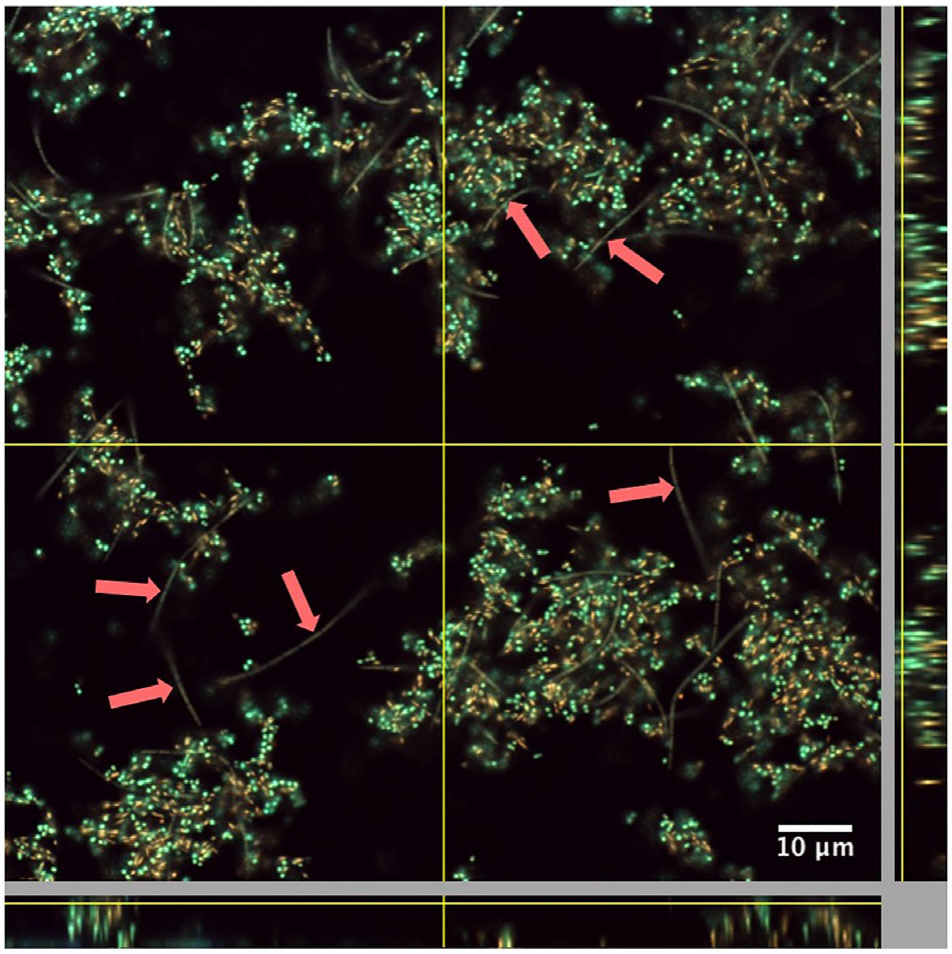
CLSM image of a fixed “five‐species” biofilm containing *T. serpentiformis* and *P. gingivalis*. Yellow/red: FISH‐stained *P. gingivalis* cells; green/teal: nonhybridized cells (DNA staining Sytox green). *Tannerella serpentiformis* cells can be distinguished by their snake‐like morphology; red arrows indicate exemplarily *T. serpentiformis* cells. Objective 100×.

Based on the growth‐promoting effect of *P. gingivalis* on *T. serpentiformis*, a possible co‐localization of these bacteria was investigated in a “five‐species” biofilm containing the two bacteria, where *P. gingivalis* was FISH‐stained and *T. serpentiformis* was determined based on its distinct morphology (Figure 6). The presence of *P. gingivalis* did not alter the distribution and localization of *T. serpentiformis* in any discernible way and the two bacteria were not found to co‐localize. Nevertheless, *T. serpentiformis* was highly abundant in “five species” biofilms with *P. gingivalis* (compare with Figure 4).

### 3.6 Invasion and adhesion of *T. serpentiformis* and *T. forsythia* to Ca9‐22 cells

**FIGURE 7:**
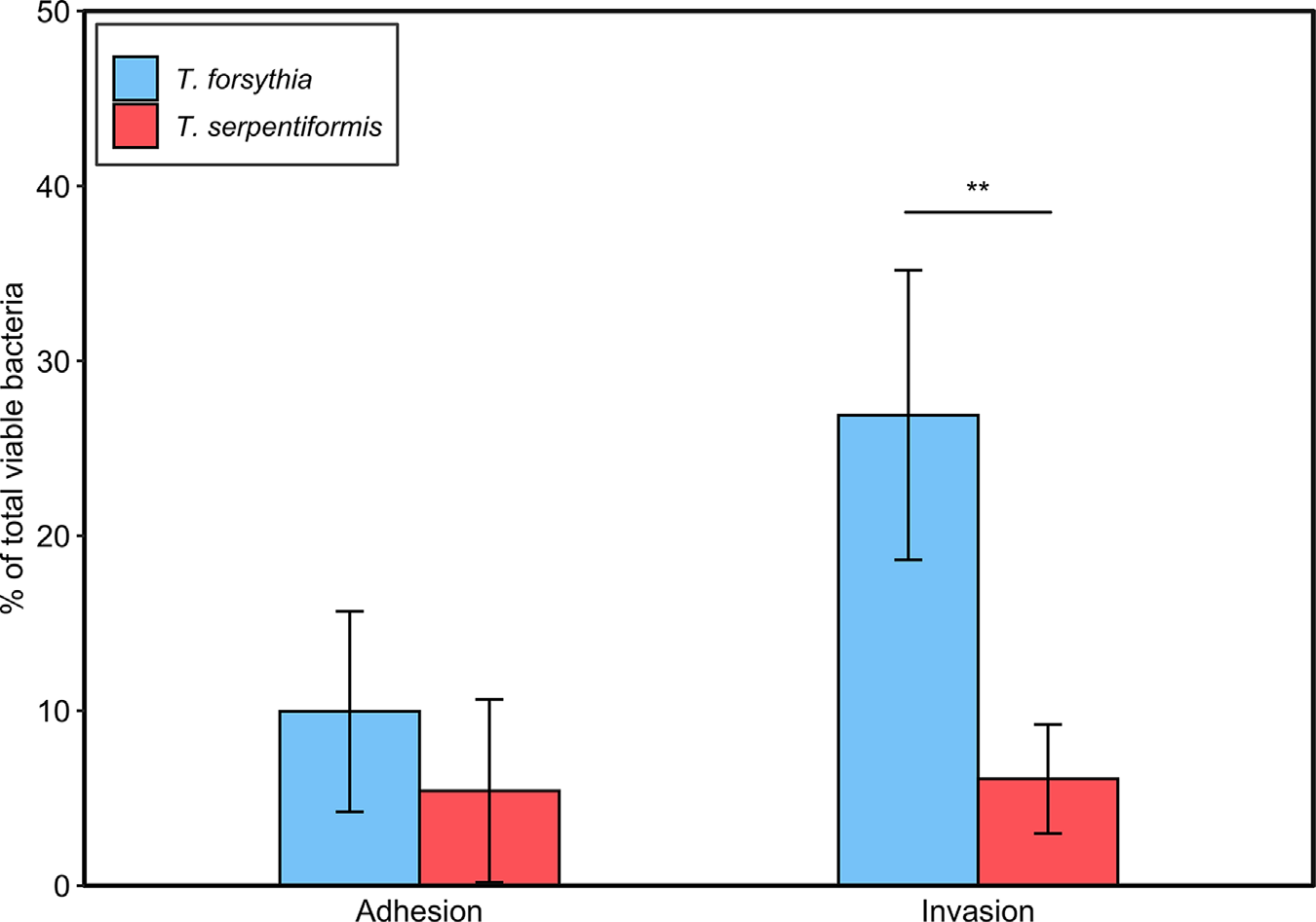
*Tannerella forsythia* (blue) and *T. serpentiformis* (red) invading or adhering to Ca9‐22 cells. Following infection at a MOI of 100, intracellular and Ca9‐22 cell‐associated bacteria were spotted in triplicate on blood agar plates and the CFUs were counted. The y‐axis shows the percentage of CFUs counted for the invaded or adherent bacteria in relation to those counted for the corresponding *Tannerella* species in cell‐free medium. Four independent experiments with three technical replicates per assay were performed. Data are presented as mean ± SD. Asterisks indicate statistically significant differences between the strains as determined by the unpaired Student's *t*‐test (**p < 0.01).

Figure 7 shows the percentage of viable intracellularly invaded and extracellularly adherent *Tannerella* species upon infection of Ca9‐22 cells. The percentage of intracellularly invaded and surviving bacteria was significantly higher for *T. forsythia* (26.90 ± 8.28%) than for *T. serpentiformis* (6.10 ± 3.12%). No significant difference in the percentage of extracellularly adherent bacteria was observed between the two *Tannerella* species, with 9.96 ± 7.73% and 5.42 ± 5.23% for *T. forsythia* and *T. serpentiformis*, respectively.

### 3.7 MTT cell viability assay

**FIGURE 8:**
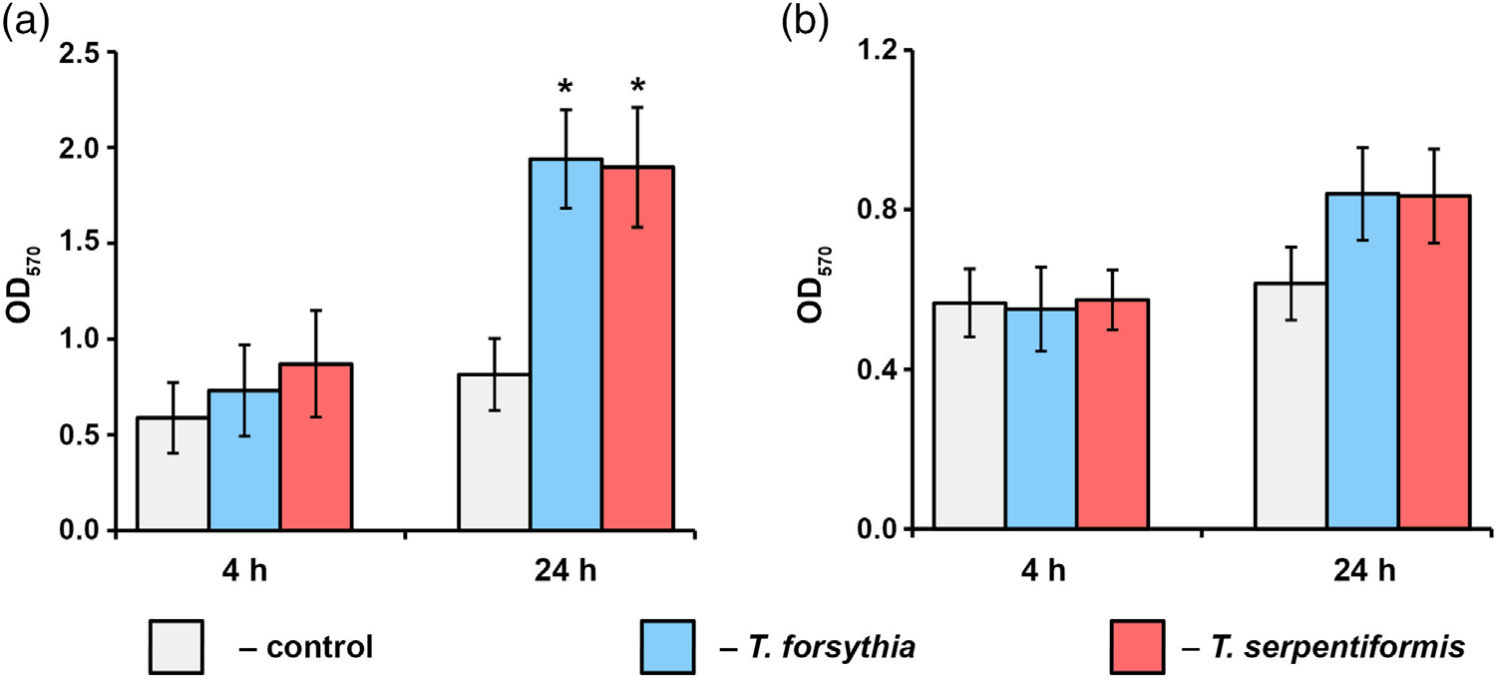
Effect of *T. forsythia* and *T. serpentiformis* on the viability of (a) U937 macrophages and (b) human gingival fibroblasts (hGFs). Cells were stimulated with *T. forsythia* or *T. serpentiformis* at a multiplicity of infection (MOI) 50 for 4 h or 24 h, and cell viability was measured by the MTT method. Cells without bacterial stimulation served as a control. The y‐axis shows OD_570_ values. Data are presented as mean ± s.e.m. of five independent experiments. *Significantly different from control.

Prior to studying the immunestimulatory potential of *T. forsythia* and *T. serpentiformis* in U937 macrophages and hGFs, the influence of the two *Tannerella* species on the viability of the human cells was determined (Figure 8). Four hours post stimulation, no significant effect of the bacteria on the viability of both host cell types was observed, while 24 h post stimulation, both *T. forsythia* and *T. serpentiformis* significantly increased the viability of U937 macrophages, but no difference between the two *Tannerella* species was found. A similar tendency was observed for hGFs, but the increase in viability was statistically insignificant.

### 3.8 Inflammatory response elicited by *T. serpentiformis* and *T. forsythia* in U937 macrophages

**FIGURE 9:**
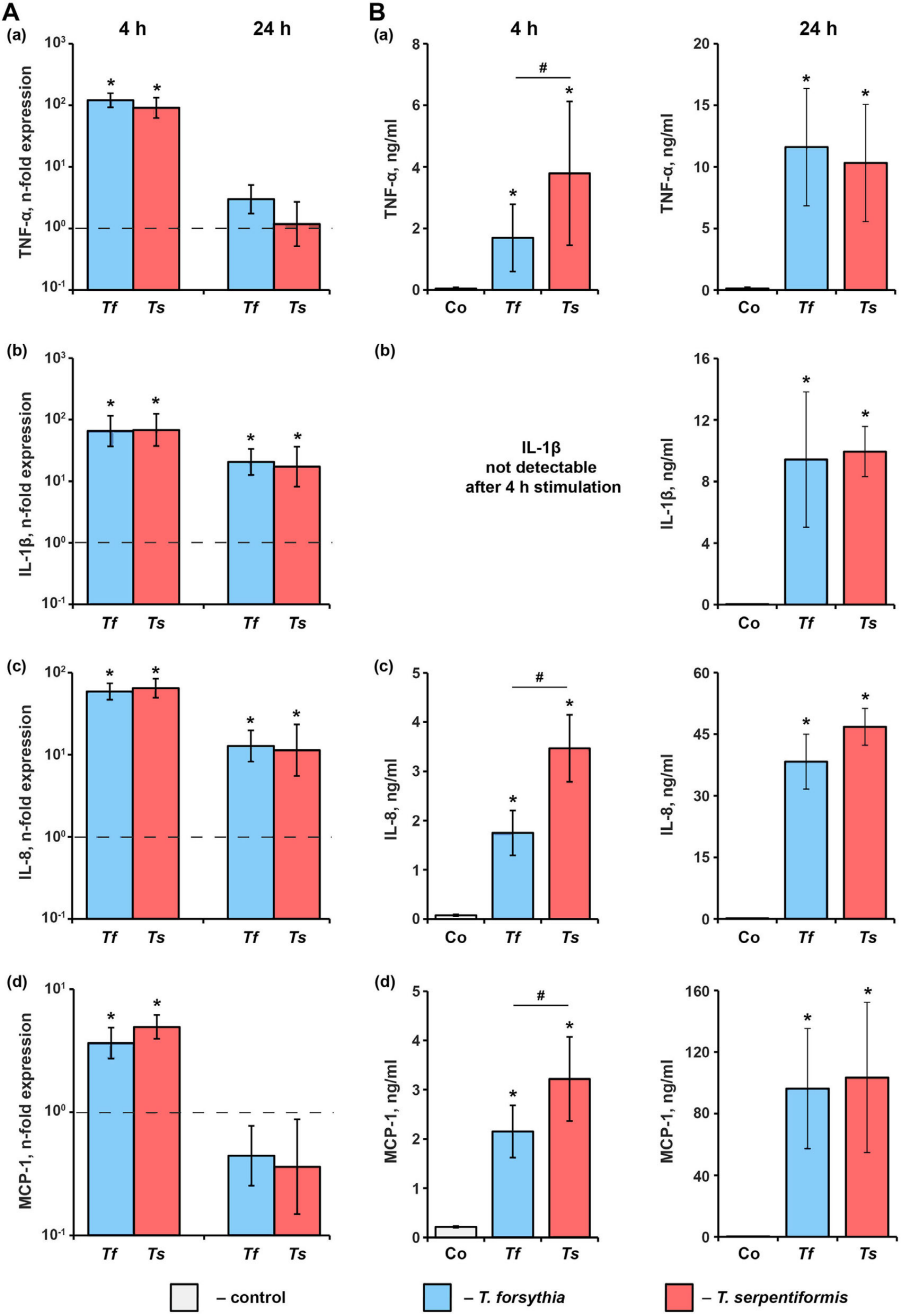
Inflammatory response of U937 macrophages upon the stimulation with *T. forsythia* and *T. serpentiformis*. U937 macrophages were stimulated with *T. forsythia* and *T. serpentiformis* at a MOI of 50 for 4 h or 24 h and (A) the resulting gene‐expression of tumor necrosis factor α (TNF‐α) (a), Interleukin‐1β (IL‐1β) (b), IL‐8 (c), and monocyte chemoattractant protein‐1 (MCP‐1) (d), and (B) the concentration of corresponding proteins in conditioned media was determined by qPCR and ELISA, respectively. Changes in the gene expression of target protein (*n*‐fold expression) was calculated by the 2^–ΔΔCt^ method taking unstimulated cells as a control (*n*‐fold expression = 1, shown by the dotted lines). Data are presented as mean ± s.e.mEM of five independent experiments. *Significantly different from control. #Significantly different between *T. forsythia* and *T. serpentiformis*.

The effect of *T. forsythia* and *T. serpentiformis* on the production of TNF‐α, IL‐1 β, IL‐8, and MCP‐1 in U937 macrophages four and 24 h post stimulation was investigated (Figure 9). Both *Tannerella* species induced a significant increase in the gene expression of all investigated inflammatory mediators after 4 h. After 24 h, significantly higher gene expression levels compared to the controls were observed for IL‐1β and IL‐8, but not for TNF‐α and MCP‐1. No significant difference in the gene expression of all investigated mediators was observed between *T. forsythia* and *T. serpentiformis* at both analyzed time points. On the protein level, IL‐1β was below the detection limit 4 h post stimulation, but otherwise, both *Tannerella* species induced a significant increase in the concentrations of all investigated inflammatory mediators. Four hours post stimulation, *T. serpentiformis* induced a significantly higher concentration of TNF‐α and IL‐8 in the conditioned media compared to *T. forsythia*, whereas no difference in the concentration of MCP‐1 was observed. IL‐1β was not detected in the conditioned media 4 h post stimulation. Twenty‐four hours post stimulation, no significant difference in the concentrations of all investigated mediators was detected.

### 3.9 Inflammatory response elicited by *T. serpentiformis* and *T. forsythia* in human gingival fibroblasts

**FIGURE 10:**
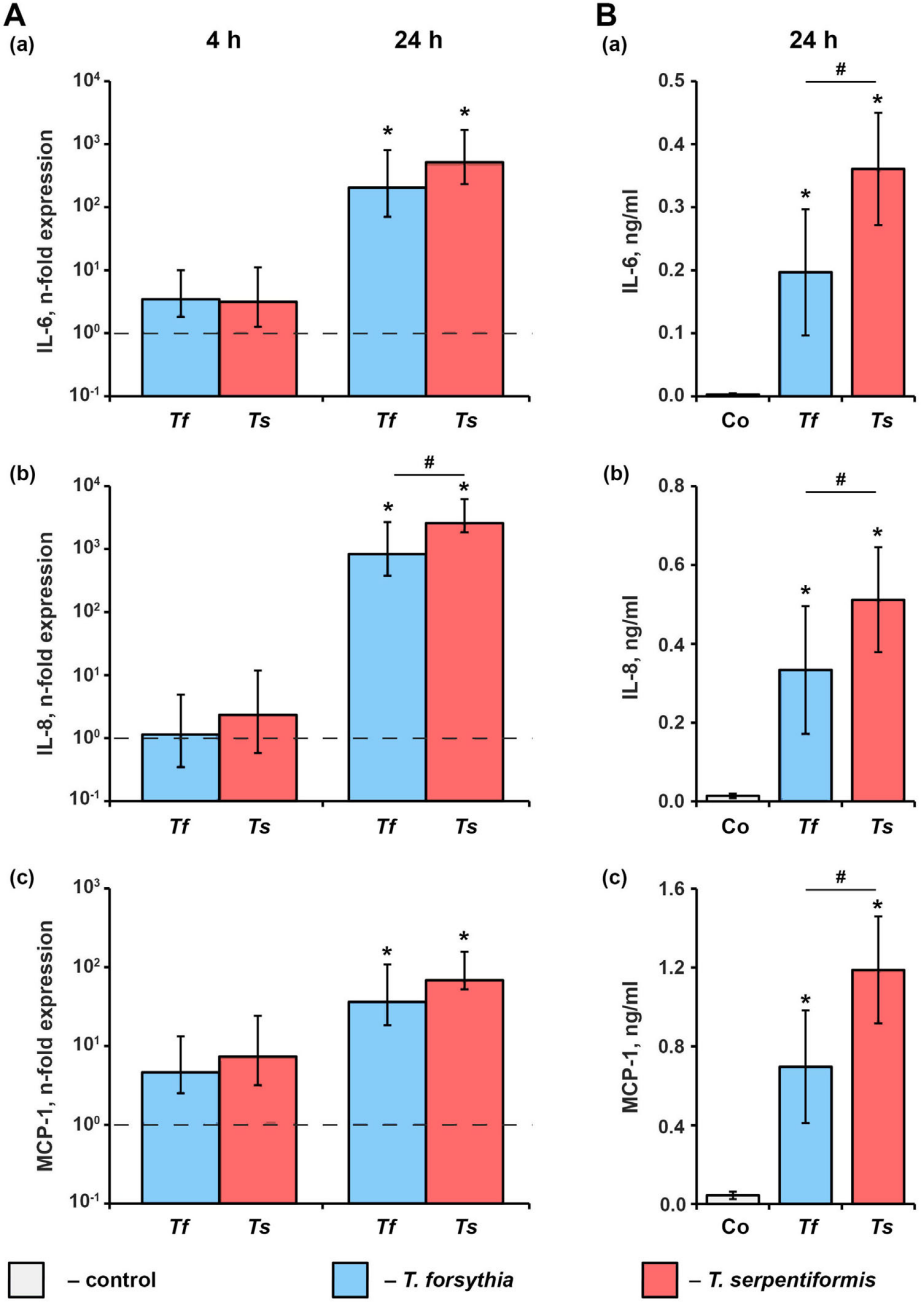
Inflammatory response of human gingival fibroblasts (hGFs) upon the stimulation with *T. forsythia* and *T. serpentiformis*. hGFs were stimulated with *T. forsythia* and *T. serpentiformis* at a multiplicity of infection (MOI) and the resulting gene‐expression of (A) interleukin 6 (IL‐6), IL‐8, and monocyte chemoattractant protein‐1 (MCP‐1) after 4 h and 24 h and (B) the concentration of corresponding proteins in conditioned media after 24 h were assessed by qPCR and ELISA, respectively. Please note that 4 h post stimulation, protein concentrations were below the detection limit. Changes in the gene expression of target protein (*n*‐fold expression) was calculated by the 2^–ΔΔCt^ method taking unstimulated cells as a control (*n*‐fold expression = 1, shown by the dotted lines). Data are presented as mean ± s.e.m. of six independent experiments with cells isolated from six different donors. *Significantly different from control. #Significantly different between *T. forsythia* and *T. serpentiformis*.

Figure 10 shows the production of IL‐6, IL‐8, and MCP‐1 by hGFs in response to the stimulation with *T. forsythia* and *T. serpentiformis* four and 24 h post stimulation. While after 4 h, no significant changes in the gene expression of the inflammatory mediators were found, both *T. forsythia* and *T. serpentiformis* induced a significant increase in the gene expression of all proteins after 24 h. *Tannerella serpentiformis* induced a significantly higher gene expression of IL‐8 than *T. forsythia*, but no difference in the gene expression of IL‐6 and MCP‐1 was observed. After 4 h, the concentration of all proteins in the conditioned media was below the detection limit. After 24 h, the levels of IL‐6, IL‐8, and MCP‐1 protein in the conditioned media were significantly increased by both bacteria, and the levels induced by *T. serpentiformis* were significantly higher than those induced by *T. forsythia*.

## 4 DISCUSSION

Biofilm formation on the tooth surface results in bacterial colonization and invasion of gingival tissue which plays an important role in the pathogenesis of periodontitis. This study was devised to compare two phylogenetically closely related bacterial species—the novel, Gram‐negative oral anaerobe *T. serpentiformis*, which is predictably associated with a healthy periodontium, and the “red‐complex” member *T. forsythia*—with regards to their behavior in a multispecies oral model biofilm and their interaction with various human host cells.

In an in vitro set‐up, the effects of the two *Tannerella* species on biofilms grown on pellicle‐coated HA discs and consisting of the five species *F. nucleatum*, *A. oris S. oralis*, *S. anginosus*, and *V. dispar* were investigated. *Porphyromonas gingivalis* was included in this study due to its documented role as a keystone pathogen and contributor to the progression of periodontitis and its potential interplay with *T. forsythia* (W. D. Zhu & Lee, 2016).

An important finding was unraveled by the simultaneous addition of the two *Tannerella* species to the multispecies biofilm, revealing a competitive effect between *T. forsythia* and *T. serpentiformis* (Figure 3). Furthermore, *P. gingivalis’* important role in the microbial consortium was supported by demonstrating its growth‐promoting effect not only on the commensal species and *F. nucleatum* but also on *T. forsythia* and *T. serpentiformis* (Figure 4). This is in accordance with the literature; *P. gingivalis* was described to cause an overall increase in the bacterial load of biofilms (Hajishengallis & Lamont, 2012), which is characteristic of periodontitis. *Tannerella serpentiformis*, but not *T. forsythia*, decreased the growth of the “five species” and mitigated *P. gingivalis*’ growth‐promoting effect, especially on *S. oralis*. Notably, in a previous study, *S. oralis* showed significantly decreased numbers in the absence of the ‘‘red complex’’ at 24 h (*p* < 0.05), followed by a significant increase at 48 h (Thurnheer et al., 2014). *Streptococcus oralis* was also found to serve as commensal keeper of homeostasis in the subgingival biofilm by antagonizing *Streptococcus mutans*, thereby preventing a caries‐favoring dysbiotic state (Thurnheer & Belibasakis, 2018). Influencing or preventing oral biofilm growth to a certain degree by *T. serpentiformis* could be an important mechanism for maintaining the symbiotic relationship between the oral microbiome and the host immune system in vivo and support the role of this species in oral health maintenance.

In contrast to *T. forsythia* (W. D. Zhu & Lee, 2016), no co‐localization or co‐aggregation of *T. serpentiformis* with *P. gingivalis* could be visualized by CLSM in biofilms after FISH‐staining or by SEM (Figure 5). This was surprising, considering that the S‐layer, which both *Tannerella* species possess as an outermost cell surface coating (Frey et al., 2018; Sekot et al., 2012), is presumed to be the major driver in co‐aggregation (W. D. Zhu & Lee, 2016). However, given that antibodies raised against the *T. forsythia* S‐layer do not cross‐react with the S‐layer of *T. serpentiformis* (Figure 1c), different epitopes might be presented at the cell surface of these bacteria. The S‐layer was also described to be important for *T. forsythia*’s monospecies biofilm formation (Bloch et al., 2017a). Biofilm formation was, however, significantly decreased or absent for *T. serpentiformis* compared to *T. forsythia* (Figure S2). Another factor involved in this observation might be the absence of the NanH sialidase in *T. serpentiformis* (Beall et al., 2018) which was described to be important for *T. forsythia*’s biofilm formation on mucin‐covered supports (Roy et al., 2011).

Concerning the interaction capability of *T. serpentiformis* and *T. forsythia* with different host cells, a substantial difference between the two *Tannerella* species was observed in their ability to invade to and survive in epithelial Ca9‐22 cells (Figure 7). Host cell invasion is a useful mechanism for bacteria to evade immune effector molecules and utilize a nutrient‐rich environment supporting their pathogenicity (Lamont & Hajishengallis, 2015). This ability was shown previously for several putative periodontal pathogens, including *P. gingivalis*, *T. denticola*, *T. forsythia*, and *F. nucleatum* (Inagaki et al., 2016; Lamont et al., 1995; Li et al., 2015; Mishima & Sharma, 2011; Zhang et al., 2021). Our data show that the invasion and survival capability of *T. serpentiformis* in oral epithelial cells is markedly lower than that of *T. forsythia*, underlining the association of *T. serpentiformis* with oral health. The difference between *T. serpentiformis* and *T. forsythia* in their invasive capability might involve the NanH sialidase, considering that this enzyme is absent in *T. serpentiformis*, and NanH‐deficient *T. forsythia* exhibited impaired epithelial cell invasion compared to the wild‐type bacterium (Honma et al., 2011).

Both *T. serpentiformis* and *T. forsythia* had no detrimental effect on the viability of U937 macrophages and primary hGFs (Figure 8). Moreover, both *Tannerella* species significantly increased the viability of U937 macrophages—with no significant difference observed between them—and did not affect that of hGFs. A similar observation regarding cell viability was made in one of our recent studies (Braun et al., 2022). An increased viability of U937 macrophages upon infection with *Tannerella* species could be due to metabolic remodeling of these cells after bacterial infection (Fleetwood et al., 2017). However, the implications on oral health maintenance and progression of periodontitis are unclear.

*Tannerella serpentiformis* induced generally a higher production of various pro‐inflammatory mediators by host cells than *T. forsythia*. Particularly, a significant difference was observed for TNF‐α, IL‐8, and MCP‐1 four h post‐infection (Figure 9), and of IL‐6, IL‐8, and MCP‐1 24 h post‐infection (Figure 10) in U937 macrophages and hGFBs, respectively. The production of inflammatory mediators by the host cells upon bacterial stimulation is most probably mediated through various pattern recognition receptors, particularly toll‐like receptor (TLR) family members, which recognize different pathogen‐associated molecular patterns (PAMPs) (Underhill & Ozinsky, 2002). Specifically, TLR‐2 recognizes peptidoglycan and lipoproteins as PAMPs, whereas TLR‐4 recognizes lipopolysaccharides (LPS) of Gram‐negative bacteria (Behm et al., 2020; Di Lorenzo et al., 2020; Nativel et al., 2017). For *P. gingivalis* LPS, TLR‐4 signaling was only recently confirmed (Nativel et al., 2017), while it was previously assumed that *P. gingivalis* LPS can act as a TLR2 or TLR4 agonist, depending on the TLR expression of the host cell, that is, TLR‐4 on endothelial cells versus TLR‐2 on epithelial cells (Kocgozlu et al., 2009). Regarding this present work, it is important to note it was previously shown that U937 macrophages as well as hGFBs express both TLR‐2 and TLR‐4 (Andrukhov, 2021; Jin et al., 2011). Determining the distinct contribution of TLRs to the host response to different *Tannerella* species remains an important subject of future studies. Additionally, differences in S‐layer glycosylation of the investigated *Tannerella* species might be of immunological relevance. There are indications that Mincle (Macrophage inducible C‐type lectin) recognizes the S‐layer glycoprotein of *T. forsythia* and thus is involved in the modulation of the cytokine response of macrophages against the bacterium (Chinthamani et al., 2017).

The immune response is a double‐edged sword: on the one hand, it is directed to eliminate overgrowing bacteria; on the other hand, it can cause collateral host tissue damages. The inflammatory mediators investigated in our study are involved in various stages of the host immune response—IL‐1β, TNF‐α, and IL‐6 promote the immune response and might directly induce tissue destruction (Palmqvist et al., 2008), whereas IL‐8 and MCP‐1 are chemokines stimulating the infiltration of neutrophils and monocytes, respectively (Baggiolini et al., 1994; Silva et al., 2007). Considering that *T. serpentiformis* is considered health‐associated and putatively balancing the oral biofilm, an increased inflammatory host response could be essential for its effective elimination in case of overgrowth. Particularly, a higher production of IL‐8 and MCP‐1 induced by *T. serpentiformis* might attract more leukocytes in vivo, which could be essential for the growth control of this bacterium, and for the biofilm growth in general.

The reasons for the higher inflammatory response to *T. serpentiformis* compared to *T. forsythia* remain to be investigated. It might involve the differences in the S‐layer and its glycosylation in the two *Tannerella* species (Figure 1c,d) (Frey et al., 2018; Tomek et al., 2018; Zwickl et al., 2020). As shown by our previous study, the S‐layer of *T. forsythia* delays the inflammatory response of macrophages and hGFs to this bacterium (Sekot et al., 2011). Furthermore, the absence of several KLIKK proteases in *T. serpentiformis*, which are considered important virulence factors of *T. forsythia* possessing proteolytic ability against several host proteins (Ksiazek et al., 2015) might play a role. Interestingly, in some cases, differences in the inflammatory response between the *Tannerella* species were observed only on the protein but not on the gene level. This is probably due to the fact that gene expression level reflects the response at a certain time point, whereas proteins accumulate in the conditioned media during the whole coculture time.

Oral health is characterized by symbiotic interactions between the oral microbiome and the host immune system, whereas periodontitis is considered a dysbiotic state (Hajishengallis et al., 2020). For a long time, subgingival biofilms were considered an important etiological factor in the development of periodontitis (Curtis et al., 2020). However, the studies of the last decade revealed that the etiology of periodontitis is highly complex involving several ecological and genetic factors (Loos & Van Dyke, 2020; Rosier et al., 2018). Although the involvement of bacterial biofilms in the initiation of periodontitis is debated (Bartold & Van Dyke, 2019), microbiological studies using 16S rRNA sequencing showed that periodontitis is associated with both a qualitative and a quantitative alteration of the oral microbiome, particularly with a higher total biomass and increased prevalence of several Gram‐negative anaerobic bacteria, including the “red‐complex” bacteria *P. gingivalis*, *T. denticola*, and *T. forsythia* (Abusleme et al., 2021; Costalonga & Herzberg, 2014). The transition from a health‐associated, symbiotic to periodontitis‐associated dysbiotic state could be driven by biofilm‐associated, host‐associated, or ecological factors (Loos & Van Dyke, 2020; Van Dyke et al., 2020), but in most cases, by combinations of those. The data of the present study showed that predictably health‐associated *T. serpentiformis* and periodontitis‐associated *T. forsythia* are markedly different regarding their behavior in a multispecies oral model biofilm and their interaction with the host immune system. Biofilm overgrowth, which is a characteristic of periodontitis, can be hindered by *T. serpentiformis*. Furthermore, *T. serpentiformis* exhibits a low invasion and survival potential in oral epithelial cells and induces a higher inflammatory response, implicating an effective control of this species by the host immune system. These findings emphasize the role of this species in periodontal health maintenance. A competitive relationship between *T. serpentiformis* and *T. forsythia* could play an important role in the transition from a symbiotic to dysbiotic state and thus the progression of periodontitis.

In summary, this study contributes to our understanding of the biological role of closely related *Tannerella* species with different pathogenicity potential, pointing toward a nonpathogenic lifestyle of *T. serpentiformis* as a member of the commensal oral biofilm consortium which may be harnessed for pathogen inhibition and future therapeutic approaches.

## CONFLICT OF INTEREST

The authors declare no conflict of interest.

## Supporting information

Supp Information

## Data Availability

The data that support the findings of this study are available from the corresponding author upon reasonable request.

## References

[omi12385-bib-0001] Abdi, K. , Chen, T. , Klein, B. A. , Tai, A. K. , Coursen, J. , Liu, X. , Skinner, J. , Periasamy, S. , Choi, Y. , Kessler, B. M. , Palmer, R. J. , Gittis, A. , Matzinger, P. , Duncan, M. J. , & Singh, N. J. (2017). Mechanisms by which *Porphyromonas gingivalis* evades innate immunity. PLoS One, 12(8), e0182164. 10.1371/journal.pone.0182164 28771533 PMC5542538

[omi12385-bib-0002] Abusleme, L. , Hoare, A. , Hong, B. Y. , & Diaz, P. I. (2021). Microbial signatures of health, gingivitis, and periodontitis. Periodontology 2000, 86(1), 57–78. 10.1111/prd.12362 33690899

[omi12385-bib-0003] Ammann, T. W. , Bostanci, N. , Belibasakis, G. N. , & Thurnheer, T. (2013). Validation of a quantitative real‐time PCR assay and comparison with fluorescence microscopy and selective agar plate counting for species‐specific quantification of an *in vitro* subgingival biofilm model. Journal of Periodontal Research, 48(4), 517–526. 10.1111/jre.12034 23278531

[omi12385-bib-0004] Ammann, T. W. , Gmür, R. , & Thurnheer, T. (2012). Advancement of the 10‐species subgingival Zurich biofilm model by examining different nutritional conditions and defining the structure of the *in vitro* biofilms. BMC Microbiology, 12, 227. 10.1186/1471-2180-12-227 23040057 PMC3561252

[omi12385-bib-0005] Andrukhov, O. (2021). Toll‐like receptors and dental mesenchymal stromal cells. Frontiers in Oral Health, 2, 648901. 10.3389/froh.2021.648901 35048000 PMC8757738

[omi12385-bib-0006] Ansbro, K. , Wade, W. G. , & Stafford, G. P. (2020). *Tannerella serpentiformis* sp. nov., isolated from the human mouth. International Journal of Systematic and Evolutionary Microbiology, 70(6), 3749–3754. 10.1099/ijsem.0.004229 32519941

[omi12385-bib-0007] Baggiolini, M. , Dewald, B. , & Moser, B. (1994). Interleukin‐8 and related chemotactic cytokines–CXC and CC chemokines. Advances in Immunology, 55, 97–179. 8304236

[omi12385-bib-0008] Bartold, P. M. , & Van Dyke, T. E. (2019). An appraisal of the role of specific bacteria in the initial pathogenesis of periodontitis. Journal of Clinical Periodontology, 46(1), 6–11. 10.1111/jcpe.13046 PMC6357965 30556922

[omi12385-bib-0009] Beall, C. J. , Campbell, A. G. , Griffen, A. L. , Podar, M. , & Leys, E. J. (2018). Genomics of the uncultivated, periodontitis‐associated bacterium *Tannerella* sp. BU045 (Oral Taxon 808). mSystems, 3(3), e00018–00018. 10.1128/mSystems.00018-18 29896567 PMC5989130

[omi12385-bib-0010] Behm, C. , Blufstein, A. , Abhari, S. Y. , Koch, C. , Gahn, J. , Schäffer, C. , Moritz, A. , Rausch‐Fan, X. , & Andrukhov, O. (2020). Response of human mesenchymal stromal cells from periodontal tissue to LPS depends on the purity but not on the LPS source. Mediators of Inflammation, 2020, 8704896. 10.1155/2020/8704896 32714091 PMC7352132

[omi12385-bib-0011] Behm, C. , Blufstein, A. , Gahn, J. , Noroozkhan, N. , Moritz, A. , Rausch‐Fan, X. , & Andrukhov, O. (2019). Soluble CD14 enhances the response of periodontal ligament stem cells to Toll‐like receptor 2 agonists. Mediators of Inflammation, 2019, 8127301. 10.1155/2019/8127301 31178663 PMC6507176

[omi12385-bib-0012] Bloch, S. , Thurnheer, T. , Murakami, Y. , Belibasakis, G. N. , & Schäffer, C. (2017a). Behavior of two *Tannerella forsythia* strains and their cell surface mutants in multispecies oral biofilms. Molecular Oral Microbiology, 32(5), 404–418. 10.1111/omi.12182 28382776 PMC5600126

[omi12385-bib-0013] Bloch, S. , Thurnheer, T. , Murakami, Y. , Belibasakis, G. N. , & Schäffer, C. (2017b). Biofilm behavior of *Tannerella forsythia* strains and S‐layer glycosylation mutants. Journal of Oral Microbiology, 9(1), 1325190. 10.1080/20002297.2017.1325190 PMC5600126 28382776

[omi12385-bib-0014] Bloch, S. , Tomek, M. B. , Friedrich, V. , Messner, P. , & Schäffer, C. (2019). Nonulosonic acids contribute to the pathogenicity of the oral bacterium *Tannerella forsythia* . Interface Focus, 9(2), 20180064. 10.1098/rsfs.2018.0064 30842870 PMC6388019

[omi12385-bib-0015] Bloch, S. , Zwicker, S. , Bostanci, N. , Sjöling, A. , Bostrom, E. A. , Belibasakis, G. N. , & Schäffer, C. (2018). Immune response profiling of primary monocytes and oral keratinocytes to different *Tannerella forsythia* strains and their cell surface mutants. Molecular Oral Microbiology, 33(2), 155–167. 10.1111/omi.12208 29235255

[omi12385-bib-0016] Blufstein, A. , Behm, C. , Gahn, J. , Uitz, O. , Naumovska, I. , Moritz, A. , Rausch‐Fan, X. , & Andrukhov, O. (2019). Synergistic effects triggered by simultaneous Toll‐like receptor‐2 and ‐3 activation in human periodontal ligament stem cells. Journal of Periodontology, 90(10), 1190–1201. 10.1002/JPER.19-0005 31049957 PMC6852053

[omi12385-bib-0017] Blufstein, A. , Behm, C. , Kubin, B. , Gahn, J. , Moritz, A. , Rausch‐Fan, X. , & Andrukhov, O. (2021). Anti‐apoptotic effects of human gingival mesenchymal stromal cells on polymorphonuclear leucocytes. Oral Disease, 28(3), 777–785. 10.1111/odi.13768 PMC9290793 33386669

[omi12385-bib-0018] Bradford, M. M. (1976). A rapid and sensitive method for the quantitation of microgram quantities of protein utilizing the principle of protein‐dye binding. Analytical Biochemistry, 72, 248–254. 10.1006/abio.1976.9999 942051

[omi12385-bib-0019] Braun, M. L. , Tomek, M. B. , Grünwald‐Gruber, C. , Nguyen, P. Q. , Bloch, S. , Potempa, J. S. , Andrukhov, O. , & Schäffer, C. (2022). Shut‐down of type IX protein secretion alters the host immune response to *Tannerella forsythia* and *Porphyromonas gingivalis* . Frontiers in Cellular and Infection Microbiology, 12, 835509. 10.3389/fcimb.2022.835509 35223555 PMC8869499

[omi12385-bib-0020] Chinthamani, S. , Settem, R. P. , Honma, K. , Kay, J. G. , & Sharma, A. (2017). Macrophage inducible C‐type lectin (Mincle) recognizes glycosylated surface (S)‐layer of the periodontal pathogen *Tannerella forsythia* . PLoS One, 12(3), e0173394. 10.1371/journal.pone.0173394 28264048 PMC5338828

[omi12385-bib-0021] Costalonga, M. , & Herzberg, M. C. (2014). The oral microbiome and the immunobiology of periodontal disease and caries. Immunology Letters , 162(2 Pt A), 22–38. 10.1016/j.imlet.2014.08.017 PMC4346134 25447398

[omi12385-bib-0022] Curtis, M. A. , Diaz, P. I. , & Van Dyke, T. E. (2020). The role of the microbiota in periodontal disease. Periodontology 2000, 83(1), 14–25. 10.1111/prd.12296 32385883

[omi12385-bib-0023] Darveau, R. P. (2010). Periodontitis: A polymicrobial disruption of host homeostasis. Nature Reviews Microbiology, 8(7), 481–490. 10.1038/nrmicro2337 20514045

[omi12385-bib-0024] de Andrade, K. Q. , Almeida‐da‐Silva, C. L. C. , & Coutinho‐Silva, R. (2019). Immunological pathways triggered by *Porphyromonas gingivalis* and *Fusobacterium nucleatum*: Therapeutic possibilities? Mediators of Inflammation, 2019, 7241312. 10.1155/2019/7241312 31341421 PMC6612971

[omi12385-bib-0025] Di Lorenzo, A. , Bolli, E. , Tarone, L. , Cavallo, F. , & Conti, L. (2020). Toll‐like receptor 2 at the crossroad between cancer cells, the immune system, and the microbiota. International Journal of Molecular Science, 21(24), 9418. 10.3390/ijms21249418 PMC7763461 33321934

[omi12385-bib-0026] Doerner, K. C. , & White, B. A. (1990). Detection of glycoproteins separated by nondenaturing polyacrylamide gel electrophoresis using the periodic acid‐Schiff stain. Analytical Biochemistry, 187(1), 147–150. http://www.ncbi.nlm.nih.gov/pubmed/2196831 2196831 10.1016/0003-2697(90)90433-a

[omi12385-bib-0027] Dominy, S. S. , Lynch, C. , Ermini, F. , Benedyk, M. , Marczyk, A. , Konradi, A. , Nguyen, M. , Haditsch, U. , Raha, D. , Griffin, C. , Holsinger, L. J. , Arastu‐Kapur, S. , Kaba, S. , Lee, A. , Ryder, M. I. , Potempa, B. , Mydel, P. , Hellvard, A. , … Potempa, J. (2019). *Porphyromonas gingivalis* in Alzheimer's disease brains: Evidence for disease causation and treatment with small‐molecule inhibitors. Science Advances, 5(1), eaau3333. 10.1126/sciadv.aau3333 30746447 PMC6357742

[omi12385-bib-0028] Ebersole, J. L. , Graves, C. L. , Gonzalez, O. A. , Dawson, D., 3rd , Morford, L. A. , Huja, P. E. , Hartsfield, J. K., Jr. , Huja, S. S. , Pandruvada, S. , & Wallet, S. M. (2016). Aging, inflammation, immunity and periodontal disease. Periodontology 2000, 72(1), 54–75. 10.1111/prd.12135 27501491

[omi12385-bib-0029] Fiorillo, L. , Cervino, G. , Laino, L. , D'Amico, C. , Mauceri, R. , Tozum, T. F. , Gaeta, M. , & Cicciù, M. (2019). *Porphyromonas gingivalis*, periodontal and systemic implications: A systematic review. Dental Journal, 7(4), 114. 10.3390/dj7040114 PMC6960968 31835888

[omi12385-bib-0030] Fleetwood, A. J. , Lee, M. K. S. , Singleton, W. , Achuthan, A. , Lee, M. C. , O'Brien‐Simpson, N. M. , Cook, A. D. , Murphy, A. J. , Dashper, S. G. , Reynolds, E. C. , & Hamilton, J. A. (2017). Metabolic remodeling, inflammasome activation, and pyroptosis in macrophages stimulated by *Porphyromonas gingivalis* and its outer membrane vesicles. Frontiers in Cellular and Infection Microbiology, 7, 351. 10.3389/fcimb.2017.00351 28824884 PMC5543041

[omi12385-bib-0031] Frey, A. M. , Ansbro, K. , Kamble, N. S. , Pham, T. K. , & Stafford, G. P. (2018). Characterisation and pure culture of putative health‐associated oral bacterium BU063 (*Tannerella* sp. HOT‐286) reveals presence of a potentially novel glycosylated S‐layer. FEMS Microbiology Letters, 365(17), fny180. 10.1093/femsle/fny180 30052903

[omi12385-bib-0032] Friedrich, V. , Gruber, C. , Nimeth, I. , Pabinger, S. , Sekot, G. , Posch, G. , Altmann, F. , Messner, P. , Andrukhov, O. , & Schäffer, C. (2015). Outer membrane vesicles of *Tannerella forsythia*: Biogenesis, composition, and virulence. Molecular Oral Microbiology, 30(6), 451–473. 10.1111/omi.12104 25953484 PMC4604654

[omi12385-bib-0033] Friedrich, V. , Janesch, B. , Windwarder, M. , Maresch, D. , Braun, M. L. , Megson, Z. A. , Vinogradov, E. , Goneau, M. F. , Sharma, A. , Altmann, F. , Messner, P. , Schoenhofen, I. C. , & Schäffer, C. (2017). *Tannerella forsythia* strains display different cell‐surface nonulosonic acids: Biosynthetic pathway characterization and first insight into biological implications. Glycobiology, 27(4), 342–357. 10.1093/glycob/cww129 27986835 PMC5378307

[omi12385-bib-0034] Gmür, R. , & Guggenheim, B. (1983). Antigenic heterogeneity of *Bacteroides intermedius* as recognized by monoclonal antibodies. Infection and Immunity, 42(2), 459–470. 10.1128/iai.42.2.459-470.1983 6196291 PMC264452

[omi12385-bib-0035] Hajishengallis, G. (2014). Immunomicrobial pathogenesis of periodontitis: Keystones, pathobionts, and host response. Trends in Immunology, 35(1), 3–11. 10.1016/j.it.2013.09.001 24269668 PMC3947349

[omi12385-bib-0036] Hajishengallis, G. (2015). Periodontitis: From microbial immune subversion to systemic inflammation. Nature Reviews in Immunology, 15(1), 30–44. 10.1038/nri3785 PMC4276050 25534621

[omi12385-bib-0037] Hajishengallis, G. , Chavakis, T. , & Lambris, J. D. (2020). Current understanding of periodontal disease pathogenesis and targets for host‐modulation therapy. Periodontology 2000, 84(1), 14–34. 10.1111/prd.12331 32844416 PMC7457922

[omi12385-bib-0038] Hajishengallis, G. , & Diaz, P. I. (2020). *Porphyromonas gingivalis*: Immune subversion activities and role in periodontal dysbiosis. Current Oral Health Reports, 7(1), 12–21. 10.1007/s40496-020-00249-3 33344104 PMC7747940

[omi12385-bib-0039] Hajishengallis, G. , & Lamont, R. J. (2012). Beyond the red complex and into more complexity: The polymicrobial synergy and dysbiosis (PSD) model of periodontal disease etiology. Molecular Oral Microbiology, 27(6), 409–419. 10.1111/j.2041-1014.2012.00663.x 23134607 PMC3653317

[omi12385-bib-0040] Holt, S. C. , & Ebersole, J. L. (2005). *Porphyromonas gingivalis*, *Treponema denticola*, and *Tannerella forsythia*: The “red complex”, a prototype polybacterial pathogenic consortium in periodontitis. Periodontoogyl 2000, 38, 72–122. 10.1111/j.1600-0757.2005.00113.x 15853938

[omi12385-bib-0041] Honma, K. , Mishima, E. , & Sharma, A. (2011). Role of *Tannerella forsythia* NanH sialidase in epithelial cell attachment. Infection and Immunity, 79(1), 393–401. 10.1128/IAI.00629-10 21078857 PMC3019913

[omi12385-bib-0042] Hottmann, I. , Borisova, M. , Schäffer, C. , & Mayer, C. (2021). Peptidoglycan salvage enables the periodontal pathogen *Tannerella forsythia* to survive within the oral microbial community. Microbial Physiology, 31, 123–134. 10.1159/000516751 34107471

[omi12385-bib-0043] Hottmann, I. , Mayer, V. M. T. , Tomek, M. B. , Friedrich, V. , Calvert, M. B. , Titz, A. , Schäffer, C. , & Mayer, C. (2018). *N*‐Acetylmuramic acid (MurNAc) auxotrophy of the oral pathogen *Tannerella forsythia*: Characterization of a MurNAc kinase and analysis of its role in cell wall metabolism. Frontiers in Microbiology, 9, 19. 10.3389/fmicb.2018.00019 29434575 PMC5790795

[omi12385-bib-0044] Inagaki, S. , Kimizuka, R. , Kokubu, E. , Saito, A. , & Ishihara, K. (2016). *Treponema denticola* invasion into human gingival epithelial cells. Microbial Pathogenesis, 94, 104–111. 10.1016/j.micpath.2016.01.010 26806000

[omi12385-bib-0045] Jin, J. , Samuvel, D. J. , Zhang, X. , Li, Y. , Lu, Z. , Lopes‐Virella, M. F. , & Huang, Y. (2011). Coactivation of TLR4 and TLR2/6 coordinates an additive augmentation on IL‐6 gene transcription via p38MAPK pathway in U937 mononuclear cells. Molecular Immunology, 49(3), 423–432. 10.1016/j.molimm.2011.08.026 22030478 PMC3224151

[omi12385-bib-0046] Kinane, D. F. , Stathopoulou, P. G. , & Papapanou, P. N. (2017). Periodontal diseases. Nature Reviews Disease Primers, 3, 17038. 10.1038/nrdp.2017.38 28805207

[omi12385-bib-0047] Kocgozlu, L. , Elkaim, R. , Tenenbaum, H. , & Werner, S. (2009). Variable cell responses to *P. gingivalis* lipopolysaccharide. Journal of Dental Research, 88(8), 741–745. 10.1177/0022034509341166 19734462

[omi12385-bib-0048] Ksiazek, M. , Mizgalska, D. , Eick, S. , Thøgersen, I. B. , Enghild, J. J. , & Potempa, J. (2015). KLIKK proteases of *Tannerella forsythia*: Putative virulence factors with a unique domain structure. Frontiers in Microbiology, 6, 312. 10.3389/fmicb.2015.00312 25954253 PMC4404884

[omi12385-bib-0049] Laemmli, U. K. (1970). Cleavage of structural proteins during the assembly of the head of bacteriophage T4. Nature, 227(5259), 680–685. 10.1038/227680a0 5432063

[omi12385-bib-0050] Lamont, R. J. , Chan, A. , Belton, C. M. , Izutsu, K. T. , Vasel, D. , & Weinberg, A. (1995). *Porphyromonas gingivalis* invasion of gingival epithelial cells. Infection and Immunity, 63(10), 3878–3885. 10.1128/iai.63.10.3878-3885.1995 7558295 PMC173546

[omi12385-bib-0051] Lamont, R. J. , & Hajishengallis, G. (2015). Polymicrobial synergy and dysbiosis in inflammatory disease. Trends in Molecular Medicine, 21(3), 172–183. 10.1016/j.molmed.2014.11.004 25498392 PMC4352384

[omi12385-bib-0052] Li, Y. , Guo, H. , Wang, X. , Lu, Y. , Yang, C. , & Yang, P. (2015). Coinfection with *Fusobacterium nucleatum* can enhance the attachment and invasion of *Porphyromonas gingivalis* or *Aggregatibacter actinomycetemcomitans* to human gingival epithelial cells. Archives in Oral Biology, 60(9), 1387–1393. 10.1016/j.archoralbio.2015.06.017 26143497

[omi12385-bib-0053] Loos, B. G. , & Van Dyke, T. E. (2020). The role of inflammation and genetics in periodontal disease. Periodontology 2000, 83(1), 26–39. 10.1111/prd.12297 32385877 PMC7319430

[omi12385-bib-0054] Marsh, P. D. , & Zaura, E. (2017). Dental biofilm: Ecological interactions in health and disease. Journal of Clinical Periodontology, 44(Suppl 18), S12–S22. 10.1111/jcpe.12679 28266111

[omi12385-bib-0055] Mayer, V. M. T. , Tomek, M. B. , Figl, R. , Borisova, M. , Hottmann, I. , Blaukopf, M. , Altmann, F. , Mayer, C. , & Schäffer, C. (2020). Utilization of different MurNAc sources by the oral pathogen *Tannerella forsythia* and role of the inner membrane transporter AmpG. BMC Microbiology, 20(1), 352. 10.1186/s12866-020-02006-z 33203363 PMC7670621

[omi12385-bib-0056] Megson, Z. A. , Koerdt, A. , Schuster, H. , Ludwig, R. , Janesch, B. , Frey, A. , Naylor, K. , Wilson, I. , Stafford, G. P. , Messner, P. , & Schäffer, C. (2015). Characterization of an α‐L‐fucosidase from the periodontal pathogen *Tannerella forsythia* . Virulence, 6(3), 282–292. 10.1080/21505594.2015.1010982 25831954 PMC4413431

[omi12385-bib-0057] Metzger, Z. , Lin, Y. Y. , Dimeo, F. , Ambrose, W. W. , Trope, M. , & Arnold, R. R. (2009). Synergistic pathogenicity of *Porphyromonas gingivalis* and *Fusobacterium nucleatum* in the mouse subcutaneous chamber model. Journal of Endodontics, 35(1), 86–94. 10.1016/j.joen.2008.10.015 19084132

[omi12385-bib-0058] Mishima, E. , & Sharma, A. (2011). *Tannerella forsythia* invasion in oral epithelial cells requires phosphoinositide 3‐kinase activation and clathrin‐mediated endocytosis. Microbiology (Reading, England), 157(Pt 8), 2382–2391. 10.1099/mic.0.048975-0 21622527 PMC3167883

[omi12385-bib-0059] Naginyte, M. , Do, T. , Meade, J. , Devine, D. A. , & Marsh, P. D. (2019). Enrichment of periodontal pathogens from the biofilms of healthy adults. Scientific Reports, 9(1), 5491. 10.1038/s41598-019-41882-y 30940882 PMC6445289

[omi12385-bib-0060] Nativel, B. , Couret, D. , Giraud, P. , Meilhac, O. , d'Hellencourt, C. L. , Viranaïcken, W. , & Da Silva, C. R. (2017). *Porphyromonas gingivalis* lipopolysaccharides act exclusively through TLR4 with a resilience between mouse and human. Scientific Reports, 7(1), 15789. 10.1038/s41598-017-16190-y 29150625 PMC5693985

[omi12385-bib-0061] Ng, H. M. , Slakeski, N. , Butler, C. A. , Veith, P. D. , Chen, Y. Y. , Liu, S. W. , Hoffmann, B. , Dashper, S. G. , & Reynolds, E. C. (2019). The Role of *Treponema denticola* motility in synergistic biofilm formation with *Porphyromonas gingivalis* . Frontiers in Cellular and Infection Microbiology, 9, 432. 10.3389/fcimb.2019.00432 31921707 PMC6930189

[omi12385-bib-0062] Oh, Y. J. , Sekot, G. , Duman, M. , Chtcheglova, L. , Messner, P. , Peterlik, H. , Schäffer, C. , & Hinterdorfer, P. (2013). Characterizing the S‐layer structure and anti‐S‐layer antibody recognition on intact *Tannerella forsythia* cells by scanning probe microscopy and small angle X‐ray scattering. Journal of Molecular Recognition, 26(11), 542–549. 10.1002/jmr.2298 24089361 PMC4397952

[omi12385-bib-0063] Onishi, S. , Honma, K. , Liang, S. , Stathopoulou, P. , Kinane, D. , Hajishengallis, G. , & Sharma, A. (2008). Toll‐like receptor 2‐mediated interleukin‐8 expression in gingival epithelial cells by the *Tannerella forsythia* leucine‐rich repeat protein BspA. Infection and Immunity, 76(1), 198–205. 10.1128/iai.01139-07 17967853 PMC2223669

[omi12385-bib-0064] Orth, R. K. , O'Brien‐Simpson, N. M. , Dashper, S. G. , & Reynolds, E. C. (2011). Synergistic virulence of *Porphyromonas gingivali*s and *Treponema denticola* in a murine periodontitis model. Molecular Oral Microbiology, 26(4), 229–240. 10.1111/j.2041-1014.2011.00612.x 21729244

[omi12385-bib-0065] Palmqvist, P. , Lundberg, P. , Lundgren, I. , Hänström, L. , & Lerner, U. H. (2008). IL‐β and TNF‐α regulate IL‐6‐type cytokines in gingival fibroblasts. Journal of Dental Research, 87(6), 558–563. 10.1177/154405910808700614 18502965

[omi12385-bib-0066] Park, D.‐G. , Woo, B. H. , Lee, B.‐J. , Yoon, S. , Cho, Y. , Kim, Y.‐D. , Park, H. R. , & Song, J. M. (2019). Serum levels of interleukin‐6 and titers of antibodies against *Porphyromonas gingivalis* could be potential biomarkers for the diagnosis of oral squamous cell carcinoma. International Journal of Molecular Sciences, 20(11), 2749. 10.3390/ijms20112749 31167516 PMC6600294

[omi12385-bib-0067] Polak, D. , Shapira, L. , Weiss, E. I. , & Houri‐Haddad, Y. (2012). The role of coaggregation between *Porphyromonas gingivali*s and *Fusobacterium nucleatum* on the host response to mixed infection. Journal of Clinical Periodontology, 39(7), 617–625. 10.1111/j.1600-051X.2012.01889.x 22607053

[omi12385-bib-0068] Posch, G. , Pabst, M. , Brecker, L. , Altmann, F. , Messner, P. , & Schäffer, C. (2011). Characterization and scope of S‐layer protein *O*‐glycosylation in *Tannerella forsythia* . Journal of Biological Chemistry, 286(44), 38714–38724. 10.1074/jbc.M111.284893 21911490 PMC3207478

[omi12385-bib-0069] Rosier, B. T. , Marsh, P. D. , & Mira, A. (2018). Resilience of the oral microbiota in health: Mechanisms that prevent dysbiosis. Journal of Dental Research, 97(4), 371–380. 10.1177/0022034517742139 29195050

[omi12385-bib-0070] Roy, S. , Honma, K. , Douglas, C. W. , Sharma, A. , & Stafford, G. P. (2011). Role of sialidase in glycoprotein utilization by *Tannerella forsythia* . Microbiology (Reading, England), 157(Pt 11), 3195–3202. 10.1099/mic.0.052498-0 21885482 PMC3352272

[omi12385-bib-0071] Sakakibara, J. , Nagano, K. , Murakami, Y. , Higuchi, N. , Nakamura, H. , Shimozato, K. , & Yoshimura, F. (2007). Loss of adherence ability to human gingival epithelial cells in S‐layer protein‐deficient mutants of *Tannerella forsythensis* . Microbiology (Reading, England), 153(3), 866–876. 10.1099/mic.0.29275-0 17322207

[omi12385-bib-0072] Schindelin, J. , Arganda‐Carreras, I. , Frise, E. , Kaynig, V. , Longair, M. , Pietzsch, T. , Preibisch, S. , Rueden, C. , Saalfeld, S. , Schmid, B. , Tinevez, J. Y. , White, D. J. , Hartenstein, V. , Eliceiri, K. , Tomancak, P. , & Cardona, A. (2012). Fiji: An open‐source platform for biological‐image analysis. Nature Methods, 9(7), 676–682. 10.1038/nmeth.2019 22743772 PMC3855844

[omi12385-bib-0073] Sekot, G. , Posch, G. , Messner, P. , Matejka, M. , Rausch‐Fan, X. , Andrukhov, O. , & Schäffer, C. (2011). Potential of the *Tannerella forsythia* S‐layer to delay the immune response. Journal of Dental Research, 90(1), 109–114. 10.1177/0022034510384622 20929722 PMC4382719

[omi12385-bib-0074] Sekot, G. , Posch, G. , Oh, Y. J. , Zayni, S. , Mayer, H. F. , Pum, D. , Messner, P. , Hinterdorfer, P. , & Schäffer, C. (2012). Analysis of the cell surface layer ultrastructure of the oral pathogen *Tannerella forsythia* . Archives in Microbiology, 194(6), 525–539. 10.1007/s00203-012-0792-3 PMC3354324 22273979

[omi12385-bib-0075] Settem, R. P. , Honma, K. , Nakajima, T. , Phansopa, C. , Roy, S. , Stafford, G. P. , & Sharma, A. (2013). A bacterial glycan core linked to surface (S)‐layer proteins modulates host immunity through Th17 suppression. Mucosal Immunology, 6(2), 415–426. 10.1038/mi.2012.85 22968422 PMC4049606

[omi12385-bib-0076] Sharma, A. , Sojar, H. T. , Glurich, I. , Honma, K. , Kuramitsu, H. K. , & Genco, R. J. (1998). Cloning, expression, and sequencing of a cell surface antigen containing a leucine‐rich repeat motif from *Bacteroides forsythus* ATCC 43037. Infection and Immunity, 66(12), 5703–5710. 10.1128/IAI.66.12.5703-5710.1998 9826345 PMC108721

[omi12385-bib-0077] Silva, T. A. , Garlet, G. P. , Fukada, S. Y. , Silva, J. S. , & Cunha, F. Q. (2007). Chemokines in oral inflammatory diseases: Apical periodontitis and periodontal disease. Journal of Dental Research, 86(4), 306–319. http://www.ncbi.nlm.nih.gov/pubmed/17384024 17384024 10.1177/154405910708600403

[omi12385-bib-0078] Socransky, S. , Haffajee, A. , Cugini, M. , Smith, C. , & Kent, R. (1998). Microbial complexes in subgingival plaque. Journal of Clinical Periodontology, 25(2), 134–144. 10.1111/j.1600-051X.1998.tb02419.x 9495612

[omi12385-bib-0079] Stafford, G. , Roy, S. , Honma, K. , & Sharma, A. (2012). Sialic acid, periodontal pathogens and *Tannerella forsythia*: Stick around and enjoy the feast! Molecular Oral Microbiology, 27(1), 11–22. 10.1111/j.2041-1014.2011.00630.x 22230462 PMC4049603

[omi12385-bib-0080] Thurnheer, T. , & Belibasakis, G. N. (2018). *Streptococcus oralis* maintains homeostasis in oral biofilms by antagonizing the cariogenic pathogen *Streptococcus mutans* . Molecular Oral Microbiology, 33(3), 234–239. 10.1111/omi.12216 29327482

[omi12385-bib-0081] Thurnheer, T. , Belibasakis, G. N. , & Bostanci, N. (2014). Colonisation of gingival epithelia by subgingival biofilms *in vitro*: Role of “red complex” bacteria. Archives in Oral Biology, 59(9), 977–986. 10.1016/j.archoralbio.2014.05.023 24949828

[omi12385-bib-0082] Thurnheer, T. , Gmür, R. , & Guggenheim, B. (2004). Multiplex FISH analysis of a six‐species bacterial biofilm. Journal of Microbiological Methods, 56(1), 37–47. 10.1016/j.mimet.2003.09.003 14706749

[omi12385-bib-0083] Thurnheer, T. , van der Ploeg, J. R. , Giertsen, E. , & Guggenheim, B. (2006). Effects of *Streptococcus mutans gtfC* deficiency on mixed oral biofilms *in vitro* . Caries Research, 40(2), 163–171. 10.1159/000091065 16508276

[omi12385-bib-0084] Tomek, M. B. , Maresch, D. , Windwarder, M. , Friedrich, V. , Janesch, B. , Fuchs, K. , Neumann, L. , Nimeth, I. , Zwickl, N. F. , Dohm, J. C. , Everest‐Dass, A. , Kolarich, D. , Himmelbauer, H. , Altmann, F. , & Schäffer, C. (2018). A general protein *O*‐glycosylation gene cluster encodes the species‐specific glycan of the oral pathogen *Tannerella forsythia*: *O*‐glycan biosynthesis and immunological implications. Frontiers in Microbiology, 9, 2008. 10.3389/fmicb.2018.02008 30210478 PMC6120980

[omi12385-bib-0085] Trindade, F. , Oppenheim, F. G. , Helmerhorst, E. J. , Amado, F. , Gomes, P. S. , & Vitorino, R. (2014). Uncovering the molecular networks in periodontitis. Proteomics Clinical Applications, 8(9–10), 748–761. 10.1002/prca.201400028 24828325 PMC4426160

[omi12385-bib-0086] Underhill, D. M. , & Ozinsky, A. (2002). Toll‐like receptors: Key mediators of microbe detection. Current Opinion in Immunology, 14(1), 103–110. 10.1016/s0952-7915(01)00304-1 11790539

[omi12385-bib-0087] Van Dyke, T. E. , Bartold, P. M. , & Reynolds, E. C. (2020). The nexus between periodontal inflammation and dysbiosis. Frontiers in Immunology, 11, 511. 10.3389/fimmu.2020.00511 32296429 PMC7136396

[omi12385-bib-0088] Vartoukian, S. R. , Adamowska, A. , Lawlor, M. , Moazzez, R. , Dewhirst, F. E. , & Wade, W. G. (2016). In vitro cultivation of ‘unculturable’ oral bacteria, facilitated by community culture and media supplementation with siderophores. PLoS One, 11(1), e0146926. 10.1371/journal.pone.0146926 26764907 PMC4713201

[omi12385-bib-0089] Vartoukian, S. R. , Moazzez, R. V. , Paster, B. J. , Dewhirst, F. E. , & Wade, W. G. (2016). First cultivation of health‐associated *Tannerella* sp. HOT‐286 (BU063). Journal of Dental Research, 95(11), 1308–1313. 10.1177/0022034516651078 27193146 PMC5076754

[omi12385-bib-0090] Vistica, D. T. , Skehan, P. , Scudiero, D. , Monks, A. , Pittman, A. , & Boyd, M. R. (1991). Tetrazolium‐based assays for cellular viability: A critical examination of selected parameters affecting formazan production. Cancer Research, 51(10), 2515–2520. https://www.ncbi.nlm.nih.gov/pubmed/2021931 2021931

[omi12385-bib-0091] Zhang, Z. , Liu, S. , Zhang, S. , Li, Y. , Shi, X. , Liu, D. , & Pan, Y. (2021). *Porphyromonas gingivalis* outer membrane vesicles inhibit the invasion of *Fusobacterium nucleatum* into oral epithelial cells by downregulating FadA and FomA. Journal of Periodontology, 2021, 1–11. 10.1002/jper.21-0144 PMC9415117 34458990

[omi12385-bib-0092] Zhu, W. D. , & Lee, S. W. (2016). Surface interactions between two of the main periodontal pathogens: *Porphyromonas gingivalis* and *Tannerella forsythia* . Journal of Periodontal and Implant Science, 46(1), 2–9. 10.5051/jpis.2016.46.1.2 26937289 PMC4771834

[omi12385-bib-0093] Zhu, Y. , Dashper, S. G. , Chen, Y. Y. , Crawford, S. , Slakeski, N. , & Reynolds, E. C. (2013). *Porphyromonas gingivalis* and *Treponema denticola* synergistic polymicrobial biofilm development. PLoS One, 8(8), e71727. 10.1371/journal.pone.0071727 23990979 PMC3753311

[omi12385-bib-0094] Züger, J. , Lüthi‐Schaller, H. , & Gmür, R. (2007). Uncultivated *Tannerella* BU045 and BU063 are slim segmented filamentous rods of high prevalence but low abundance in inflammatory disease‐associated dental plaques. Microbiology (Reading, England), 153(11), 3809–3816. 10.1099/mic.0.2007/010926-0 17975090

[omi12385-bib-0095] Zwickl, N. F. , Stralis‐Pavese, N. , Schäffer, C. , Dohm, J. C. , & Himmelbauer, H. (2020). Comparative genome characterization of the periodontal pathogen *Tannerella forsythia* . BMC Genomics [Electronic Resource], 21(1), 150. 10.1186/s12864-020-6535-y 32046654 PMC7014623

