# Supplementary material for: Multispecies biofilm behavior and host interaction support the association of Tannerella serpentiformis with periodontal health
Source: Mol Oral Microbiol. 2022 Aug 29;38(2):115–33. doi: 10.1111/omi.12385 (PMC10947601; doi:10.1111/omi.12385)
Supplement: Supplementary file 1 — Supp Information [file OMI-38-115-s001.docx]

*Supplementary Material*

# **Multispecies biofilm behaviour and host interaction support the association of *Tannerella serpentiformis* with periodontal health**

**Fabian L. Kendlbacher^1^, Susanne Bloch^1^, Fiona F. Hager-Mair^1^, Johanna Bacher^1^, Bettina Janesch^1^, Thomas Thurnheer^2^, Oleh Andrukhov^3^*, Christina Schäffer^1^***

^1^ *NanoGlycobiology* unit, Department of NanoBiotechnology, Universität für Bodenkultur Wien, Muthgasse 11, A-1190 Vienna, Austria

^2^ Clinic of Conservative and Preventive Dentistry, Division of Clinical Oral Microbiology and Immunology, Center of Dental Medicine, University of Zürich, Plattenstrasse 11, 8032 Zürich, Switzerland

^3^ Competence Center for Periodontal Research, University Clinic of Dentistry, Medical University of Vienna, A-1090 Vienna, Austria;

**Keywords:** Cell adhesion and invasion; biofilm composition and architecture; multispecies model biofilm; immunostimulatory potential; periodontitis; *Tannerella* species

**Running head:** Biological characterization of *Tannerella serpentiformis*

*Correspondence: *Christina Schäffer,* NanoGlycobiology *unit, Department of NanoBiotechnology, Universität für Bodenkultur Wien, Muthgasse 11, A-1190 Vienna, Austria*

*Tel: (+43) 1-47654 ext.80203; E-mail: christina.schaeffer@boku.ac.at*

*Oleh Andrukhov, Competence Center for Periodontal Research, University Clinic of Dentistry, Medical University of Vienna, A-1090 Vienna, Austria*

*Tel: (+43) 1-40070 ext. 2620; E-Mail: oleh.andrukhov@meduniwien.ac.at*

**
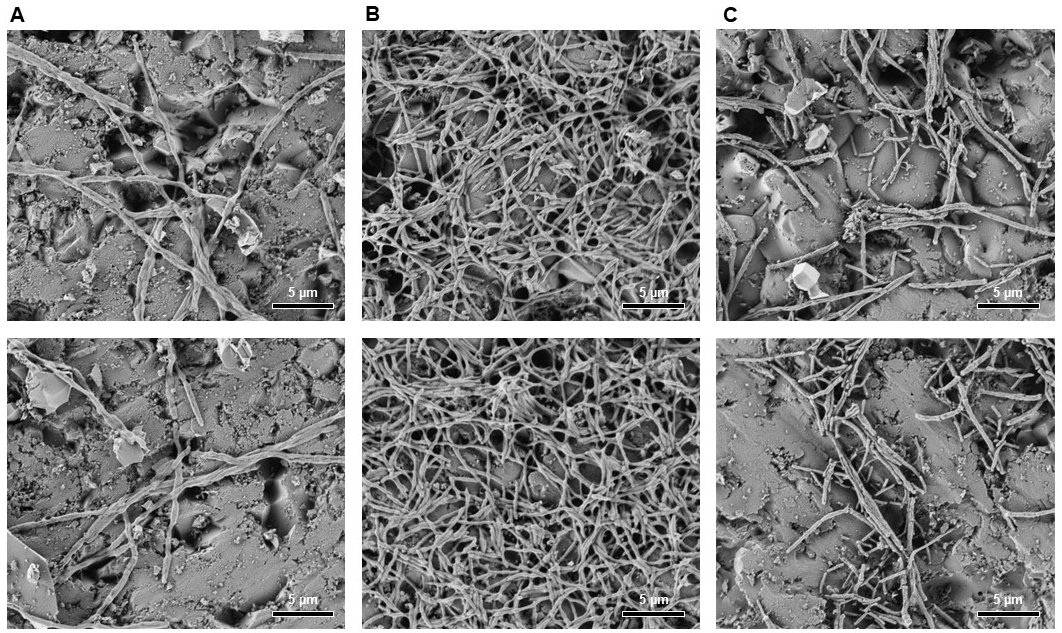
**

**FIGURE S1**Replicates of scanning electron micrographs of *T. serpentiformis* **(A)** and *T. forsythia* **(B)** cells after biofilm growth in comparison to a mixed biofilm of the two *Tannerella* sp. **(C)** grown for 64 hours on pellicle-coated HA discs. *T. forsythia* (blue arrow) and *T. serpentiformis* (red arrow) are discernible based on their cell morphology. The pictures document the reproducibility of the biofilms shown in Figure 2 of the main manuscript.

**
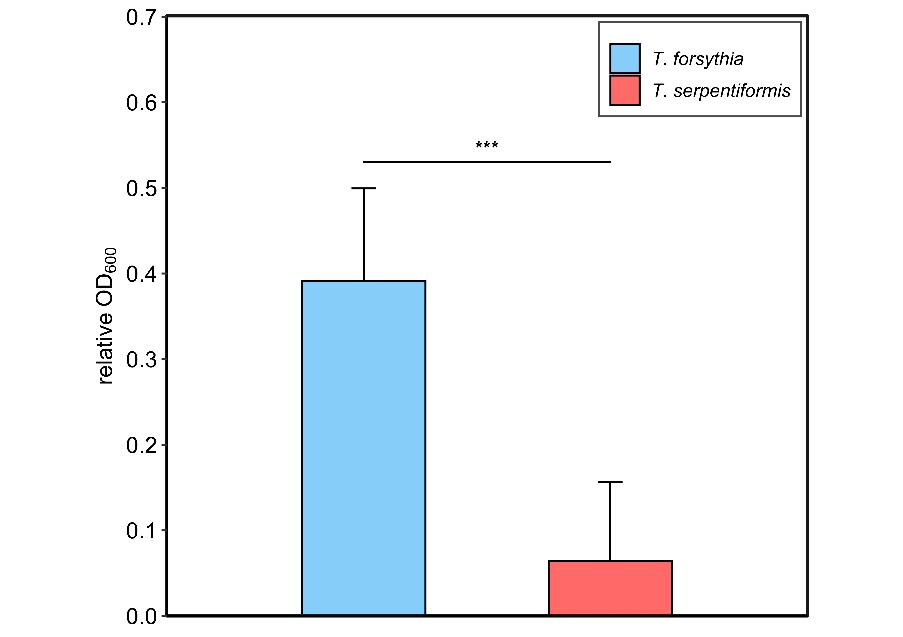
**

**FIGURE S2**Monospecies biofilm formation of *T. forsythia* (blue) and *T. serpentiformis* (red)on mucin-coated polystyrene plates. The optical densities measured for biofilms were normalized to the total OD_600_. Mean values ±SD of six independent experiments with three technical replicates each are shown. Asterisks indicate statistically significant differences between the strains as determined by the unpaired Student’s t-test (****P*< 0.001).

**
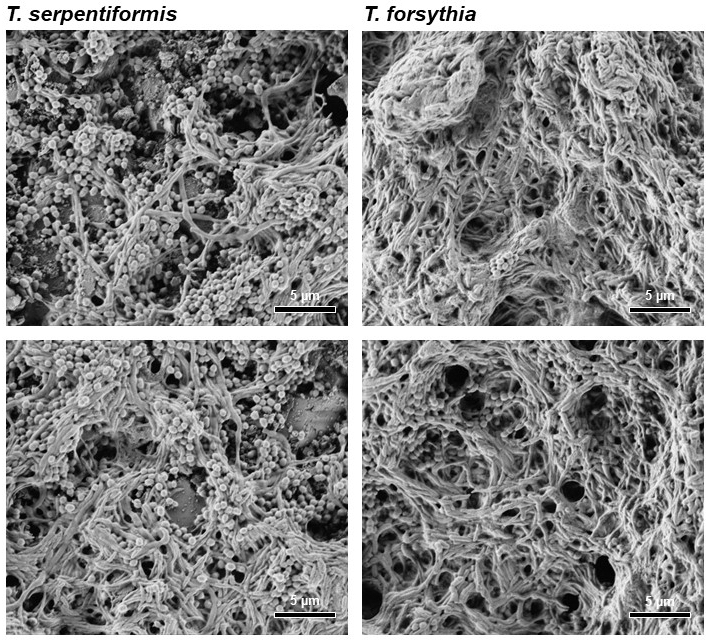
**

**FIGURE S3**Replicates of scanning electron micrographs of a fixed, “five-species” biofilm containing *T. serpentiformis* (left images) and *T. forsythia* (right images), grown for 64 hours on a pellicle-coated HA disc. The pictures document the reproducibility of the biofilms shown in Figure 5 of the main manuscript.
